# Generation of inner ear hair cells by direct lineage conversion of primary somatic cells

**DOI:** 10.7554/eLife.55249

**Published:** 2020-06-30

**Authors:** Louise Menendez, Talon Trecek, Suhasni Gopalakrishnan, Litao Tao, Alexander L Markowitz, Haoze V Yu, Xizi Wang, Juan Llamas, Chichou Huang, James Lee, Radha Kalluri, Justin Ichida, Neil Segil

**Affiliations:** 1 Department of Stem Cell and Regenerative Medicine, University of Southern CaliforniaLos AngelesUnited States; 2 Eli and Edythe Broad Center, University of Southern CaliforniaLos AngelesUnited States; 3 Zilkha Neurogenetic Institute, University of Southern CaliforniaLos AngelesUnited States; 4 USC Caruso Department of Otolaryngology – Head and Neck Surgery, University of Southern CaliforniaLos AngelesUnited States; 5 DRVision TechnologiesBellevueUnited States; Stowers Institute for Medical ResearchUnited States; The University of Hong KongHong Kong

**Keywords:** inner ear, reprogramming, sensory hair cell, screening, ototoxin, regeneration, Mouse

## Abstract

The mechanoreceptive sensory hair cells in the inner ear are selectively vulnerable to numerous genetic and environmental insults. In mammals, hair cells lack regenerative capacity, and their death leads to permanent hearing loss and vestibular dysfunction. Their paucity and inaccessibility has limited the search for otoprotective and regenerative strategies. Growing hair cells in vitro would provide a route to overcome this experimental bottleneck. We report a combination of four transcription factors (*Six1, Atoh1, Pou4f3*, and *Gfi1*) that can convert mouse embryonic fibroblasts, adult tail-tip fibroblasts and postnatal supporting cells into induced hair cell-like cells (iHCs). iHCs exhibit hair cell-like morphology, transcriptomic and epigenetic profiles, electrophysiological properties, mechanosensory channel expression, and vulnerability to ototoxin in a high-content phenotypic screening system. Thus, direct reprogramming provides a platform to identify causes and treatments for hair cell loss, and may help identify future gene therapy approaches for restoring hearing.

## eLife digest

Worldwide, hearing loss is the most common loss of sensation. Most cases of hearing loss are due to the death of specialized hair cells found deep inside the ear. These hair cells convert sounds into nerve impulses which can be understood by the brain. Hair cells naturally degrade as part of aging and can be damaged by other factors including loud noises, and otherwise therapeutic drugs, such as those used in chemotherapy for cancer. In humans and other mammals, once hair cells are lost they cannot be replaced.

Hair cells have often been studied using mice, but the small number of hair cells in their ears, and their location deep inside the skull, makes it particularly difficult to study them in this way. Scientists are seeking ways to grow hair cells in the laboratory to make it easier to understand how they work and the factors that contribute to their damage and loss. Different cell types in the body are formed in response to specific combinations of biological signals. Currently, scientists do not have an efficient way to grow hair cells in the laboratory, because the correct signals needed to create them are not known.

Menendez et al. have now identified four proteins which, when activated, convert fibroblasts, a common type of cell, into hair cells similar to those in the ear. These proteins are called Six1, Atoh1, Pou4f3 and Gfi1. Menendez et al. termed the resulting cells induced hair cells, or iHCs for short, and analyzed these cells to identify those characteristics that are similar to normal hair cells, as well as their differences. Importantly, the iHCs were found to be damaged by the same chemicals that specifically harm normal hair cells, suggesting they are useful test subjects.

The ability to create hair cells in the laboratory using more easily available cells has many uses. These cells can help to understand the normal function of hair cells and how they become damaged. They can also be used to test new drugs to assess their success in preventing or reversing hearing loss. These findings may also lead to genetic solutions to curing hearing loss.

## Introduction

Hearing loss is the most common sensory deficit with estimates of around 466 million people affected worldwide (WHO, 2019). Loss of sensory hair cells of the inner ear is the primary cause of sensorineural hearing loss (Bohne and Harding, 2000; Hinojosa et al., 2001; Géléoc and Holt, 2014; Wong and Ryan, 2015). The highly structured sensory epithelium of the inner ear, known as the organ of Corti, develops from a post-mitotic, pro-sensory domain established in the developing cochlear duct between embryonic days E12.5 and E14.5 in mice (Ruben and Sidman, 1967; Lowenheim et al., 1999; Chen and Segil, 1999; Matei et al., 2005; Lee et al., 2006). These post-mitotic cells are the progenitors for sensory hair cells and their adjacent supporting cells (Fekete et al., 1998; Kelley, 2006; Driver et al., 2013). Sensory hair cells function as the essential mechanoreceptors that convert sound vibrations into electrical signals, which are then transmitted to the brain via the spiral ganglion neurons that innervate the hair cells (Géléoc and Holt, 2003).

**Figure 1.**
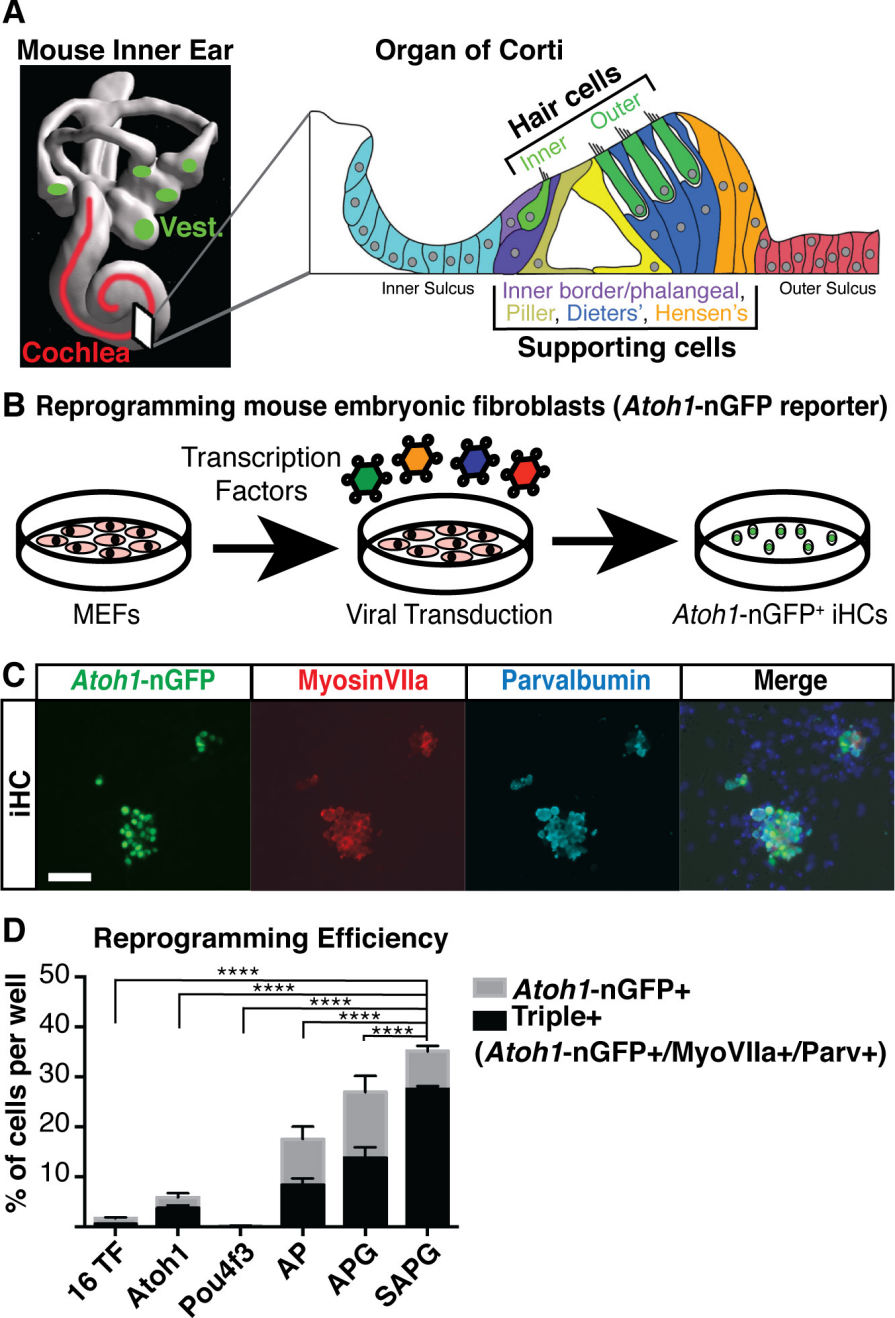
Overexpression of Six1, Atoh1, Pou4f3 and Gfi1 is capable of activating hair cell markers in mouse embryonic fibroblasts. (**A**) Diagram of the mouse inner ear shows the vestibular system (green) and the cochlea of the auditory system (red). Cross section through one turn of the cochlea shows organization in the organ of Corti as a mosaic of sensory hair cells (one row of inner hair cells and three rows of outer hair cells) interdigitated by various supporting cell populations labeled from left to right (Inner border/phalangeal, Pillar, Deiters’ and Hensen’s). Space filling model care of Steven Raft. (**B**) Schematic of experimental design for transcription factor mediated reprogramming. Mouse embryonic fibroblasts (MEFs) were isolated from *Atoh1*-nGFP transgenic reporter mice. MEFs were plated at a density of 5000 cells per well of a 96 well plate, infected with retroviral transcription factors and allowed to reprogram for 14 days prior to analysis. (**C**) Images of MEFs reprogrammed with *Six1, Atoh1, Pou4f3*, and *Gfi1* (SAPG) fixed at 14 days post infection (dpi). *Atoh1*-nGFP reporter activation (green) and immunostaining for anti-MyosinVIIa (red) and anti-Parvalbumin (cyan). Scale bar represents 50 um in length. (**D**) All quantification was performed at 14 dpi. Reprogramming efficiency was calculated as the number of *Atoh1*-nGFP positive cells divided by the 5000 MEFs plated per well. Reporter activation and immunostaining for anti-MyosinVIIa and anti-Parvalbumin was used to quantify triple positive cells (*Atoh1-*nGFP+/MyosinVIIa+/Parvalbumin+). A = *Atoh1*, P=*Pou4* f3, G = *Gfi1*, S = *Six1*. The combination SAPG gave 35% (± 1.8) reprogramming efficiency and 78% (± 1.9) of *Atoh1*-nGFP+ cells were triple positive. Statistics shown are for the comparison of triple positive cells in each condition. (N = 3 independent experiments per condition, n = 3 replicates per condition per experiment; mean ± SEM; one-way ANOVA *p<0.05, **p<0.01, ***p<0.001, ****p<0.0001).

Sensory hair cells are located in both the auditory and vestibular portions of the inner ear (Figure 1A). The hair cells within the organ of Corti are precisely arranged into one row of inner hair cells and three rows of outer hair cells, interdigitating with a variety of supporting cells; inner border, inner phalangeal, pillar cells, Deiters’ cells and Hensen’s cells (Figure 1A). Hair cells are susceptible to degeneration by a variety of genetic mutations and environmental stressors, such as exposure to loud noise, ototoxic drugs including cancer chemotherapy and aminoglycoside antibiotics, aging and over 200 known syndromic and non-syndromic genetic loci conferring predispositions to hearing loss (Matsui et al., 2004; Cheng et al., 2005; Bodmer, 2008; Langer et al., 2013; Atkinson et al., 2015; Wong and Ryan, 2015; Vaisbuch and Santa Maria, 2018). In mammals hearing and balance are dependent on the maintenance of hair cells present at birth (Groves, 2010; Géléoc and Holt, 2014), since hair cells do not spontaneously regenerate (Roberson and Rubel, 1994; Chardin and Romand, 1995; Forge et al., 1998), and so their death leads to lifelong hearing loss and balance disorders. In contrast, non-mammalian species, such as birds and reptiles, are able to spontaneously regenerate lost hair cells from existing supporting cells, leading to full functional recovery (Corwin and Cotanche, 1988; Ryals and Rubel, 1988; Stone and Cotanche, 2007; Brignull et al., 2009).

**Figure 1—figure supplement 1 .**
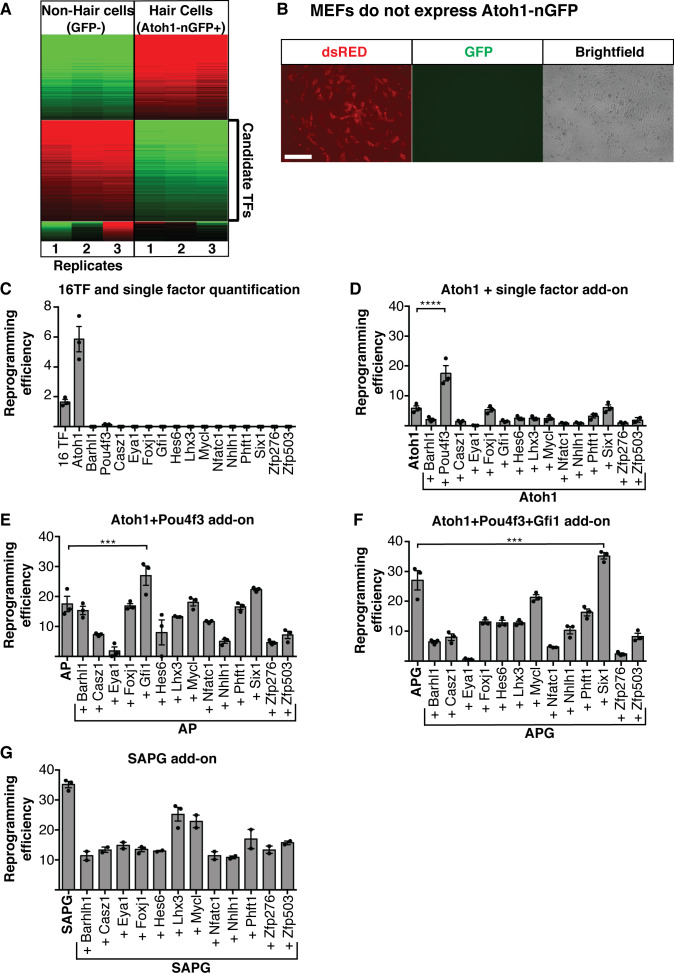
Overexpression of Six1, Atoh1, Pou4f3 and Gfi1 is capable of activating hair cell markers in mouse embryonic fibroblasts. (**A**) Heat map of RNA sequencing data from primary P1 *Atoh1*-nGFP+ cochlear hair cells compared to the *Atoh1*-nGFP- population. 16 transcription factors (TFs) were identified as only and/or highly expressed in the *Atoh1*-nGFP+ hair cell population. (n = 3 replicates per sample). (**B**) Mouse embryonic fibroblasts (MEFs) isolated from the *Atoh1*-nGFP transgenic mouse do not express the transgenic reporter. MEFs were infected with a dsRed viral control for visualization. Scale bar represents 100 um. (**C**) All quantification of reporter activation was done at 14 days post infection (dpi). Reprogramming efficiency was calculated as the number of *Atoh1*-nGFP positive cells divided by the 5000 MEFs plated per well. Reprogramming efficiency with all 16 TFs was 1.7% (± 0.3) and single factor infections only gave reporter activation with *Atoh1* alone (5.8% ± 1.5) and *Pou4f3* alone (0.15% ± 0.03). (**D**) Reprogramming efficiency with *Atoh1* alone (5.8% ± 1.5) graphed alongside single factor add-on to *Atoh1*. The addition of *Pou4f3* gave a significant increase in reprogramming efficiency to 17.5% (± 4.4). (**E**) Reprogramming efficiency with *Atoh1* and *Pou4f3* (AP, 17.5% ± 4.4) graphed alongside single factor add-on to AP. The addition of *Gfi1* gave a significant increase in reprogramming efficiency to 26.9% (± 5.6). (**F**) Reprogramming efficiency with *Atoh1, Pou4f3* and *Gfi1* (APG, 26.9% ± 5.6) graphed alongside single factor add-on to APG. The addition of *Six1* gave a significant increase in reprogramming efficiency to 35.2% (± 1.8). (**G**) Reprogramming efficiency with *Six1, Atoh1, Pou4f3*, and *Gfi1* (SAPG, 35.2% ± 1.8) graphed alongside single factor add-on to SAPG. No single factor addition gave a significant increase in reprogramming efficiency. (**C–G**): N = 3 independent experiments per condition, n = 3 replicates per condition per experiment; numbers reported as mean ± SEM; one-way ANOVA *p<0.05, **p<0.01, ***p<0.001, ****p<0.0001).

Transcription factors regulate the temporal and spatial patterns of gene expression within the cells of complex tissues, establishing cell fate, and ultimately determining their morphological and functional properties (Lemon and Tjian, 2000; Levine and Tjian, 2003; Zhang et al., 2004). Within the inner ear, expression of *Atoh1*, a bHLH class transcription factor (Lo et al., 1991; Ross et al., 2003) is both necessary and sufficient for the induction of sensory hair cells in the embryonic and neonatal cochlea, and ultimately plays an integral role in initiating the hair cell gene expression program (Bermingham et al., 1999; Zheng and Gao, 2000; Woods et al., 2004; Kelly et al., 2012; Chonko et al., 2013; Cai et al., 2013; Ryan et al., 2015; Scheffer et al., 2015; Stojanova et al., 2016; Costa et al., 2017). However, previous studies have shown that *Atoh1* expression alone is not sufficient to induce hair cell differentiation in somatic cells (Izumikawa et al., 2008; Costa et al., 2015; Abdolazimi et al., 2016), or mature supporting cells of the organ of Corti (Kelly et al., 2012; Liu et al., 2012b).

The paucity and inaccessibility of primary inner ear hair cells have limited the identification of effective otoprotective and regenerative strategies. Recent studies have demonstrated the in vitro formation of hair cells from murine pluripotent stem cells and human embryonic stem cells by directed differentiation (Oshima et al., 2010; Koehler et al., 2013; Li et al., 2003; Ronaghi et al., 2014), or in a combination of directed differentiation to an ectodermal, non-neural, placodal cell type, followed by transcription factor induction to a hair cell-like state (Costa et al., 2015). However, these elegant approaches require three-dimensional culture conditions that complicate high-throughput studies, for instance screening for otoprotectants. In contrast to morphogen-based directed differentiation of pluripotent stem cells, transcription factor (TF) -mediated lineage conversion of somatic cells enables the rapid production of neurons and other cell types in microtiter plates with ≥96 wells, allowing the reproducibility and homogeneity required for high-throughput phenotypic screening (Xu et al., 2015; Babos et al., 2019). Thus, the identification of a transcription factor cocktail that can convert somatic cells into sensory hair cells could enable screening for new otoprotective targets. Moreover, delivery of such a cocktail in vivo would enable regenerative medicine strategies for hair cell replacement in situ, which have thus far been ineffective (Izumikawa et al., 2005; Richardson and Atkinson, 2015; Roccio et al., 2015).

To this end, we have identified a cocktail of four transcription factors, *Six1*, *Atoh1*, *Pou4f3*, and *Gfi1* (SAPG), capable of converting mouse embryonic fibroblasts, adult tail tip fibroblasts, and postnatal mouse supporting cells into induced hair cells (iHCs). iHCs are highly similar to primary hair cells in terms of global gene expression and chromatin accessibility profiles, morphological features, and electrophysiological properties. In addition, we established a robotic imaging platform with automated analysis to track iHC survival and show that like primary hair cells, iHCs are selectively sensitive to gentamicin toxicity. These findings show that iHCs make a valuable in vitro model to study hair cell regeneration, maturation, function and susceptibility to ototoxins.

## Results

### Direct reprogramming of MEFs with *Six1*, *Atoh1*, *Pou4f3* and *Gfi1* activates key hair cell markers

To identify a group of TFs needed to convert somatic cells into induced hair cells, we analyzed the transcriptome of postnatal day 1 (P1) cochlear hair cells that had been FACS-purified from a transgenic mouse expressing GFP in nascent hair cells under the control of an *Atoh1* 3’ enhancer (Lumpkin et al., 2003). We compared the primary P1 cochlear hair cell transcriptome to a reference transcriptome of the FACS-purified GFP-negative cells from the same organ of Corti preparations (Figure 1—figure supplement 1A). We identified 16 candidate TFs that were highly enriched in P1 hair cells (*Atoh1*-nGFP+), some of which are known to have essential roles in hair cell development (Li et al., 2003; Wallis et al., 2003; Qian et al., 2006; Hume et al., 2007; Ahmed et al., 2012; Chonko et al., 2013; Liu et al., 2014a; Cai et al., 2015; Scheffer et al., 2015).

Using retroviral delivery, we transduced the TFs into mouse embryonic fibroblasts (MEFs) from the *Atoh1*-nGFP reporter mouse (Figure 1B). MEFs transduced with a control virus (dsRed) did not express the *Atoh1*-nGFP transgene after 14 days (Figure 1—figure supplement 1B). In contrast, overexpression of all 16 TFs led to *Atoh1*-nGFP activation in 1.7% (± 0.3) of MEFs at 14 days post infection (Figure 1—figure supplement 1C). Reprogramming efficiency was calculated as a percent of *Atoh1*-nGFP-positive MEFs out of the starting MEF number (5000 cells per well). This result indicated that within this initial group were individual transcription factors, or combinations thereof, able to reprogram MEFs to a hair cell-like state. The low level of reprogramming efficiency is expected when large numbers of factors are infected simultaneously, since only a subset of factors is expected to infect any given cell (Phan and Wodarz, 2015; Mistry et al., 2018), and since using large numbers of factors, and/or virus, is likely to challenge cellular transcription/translational machinery, thus further reducing efficiency (Babos et al., 2019).

To identify the TFs critical for the *Atoh1-*nGFP reporter activation in MEFs, we tested the efficiency of *Atoh1* and each of the other 15 TFs separately (Figure 1—figure supplement 1C). We observed that *Atoh1* alone activated the *Atoh1*-nGFP reporter in 5.8% (± 1.5) of starting MEFs, while *Pou4f3*-alone only did so in 0.15% (± 0.03) of the starting MEFs (Figure 1—figure supplement 1C). None of the other 14 factors alone activated the *Atoh1*-nGFP reporter. We then tested the reprogramming efficiency of *Atoh1* in combination with each of the other 15 TFs (Figure 1—figure supplement 1C). The most significant reporter activation came from a combination of *Atoh1* and *Pou4f3*, which provided 17.5% (± 4.4) reprogramming efficiency (Figure 1—figure supplement 1D). We then tested the addition of each remaining individual factor to the combination of *Atoh1* and *Pou4f3* (AP) (Figure 1—figure supplement 1E). *Gfi1* combined with AP (APG) increased the reporter activation to 26.9% (± 5.6) reprogramming efficiency (Figure 1—figure supplement 1E). A subsequent round of addition of individual TFs to this three-factor combination showed that the addition of *Six1* to *Atoh1*, *Pou4f3*, and *Gfi1* (SAPG) further increased the reporter activation to reach 35.2% (± 1.8) reprogramming efficiency (Figure 1—figure supplement 1F). Addition of the remaining individual factors to the cocktail of SAPG did not increase reprogramming efficiency (Figure 1—figure supplement 1G).

Since *Atoh1* is expressed in other cell types and lineages (Klisch et al., 2011; Kim et al., 2014; Ostrowski et al., 2015), we performed immunostaining for MyosinVIIa and Parvalbumin, two additional markers that are more specific to a hair cell fate (Eybalin and Ripoll, 1990; Demêmes et al., 1993; Pak and Slepecky, 1995; Hasson et al., 1997; Sahly et al., 1997; Richardson et al., 1997; Richardson et al., 1999; Boëda, 2002). The majority of SAPG-transduced cells that activated *Atoh1*-nGFP also expressed MyosinVIIa and Parvalbumin (78.4% ± 1.9) (Figure 1C,D). Overall, SAPG transduction activated *Atoh1*-nGFP with a 35% efficiency, and nearly 80% of all *Atoh1-*nGFP positive cells also expressed MyosinVIIa and Parvalbumin (Figure 1D). In contrast, only 50% of the *Atoh1*-nGFP+ cells generated by AP or APG expressed MyosinVIIa and Parvalbumin, indicating that most *Atoh1*-nGFP+ cells generated from these alternative cocktails were not hair cell-like (Figure 1D). Our results support the importance of *Six1*, *Atoh1*, *Pou4f3* and *Gfi1* in direct reprogramming of somatic cells to a hair cell-like state, with high efficiency, purity, and reproducibility.

### Induced hair cells resemble primary juvenile mouse hair cells transcriptionally

**Figure 2.**
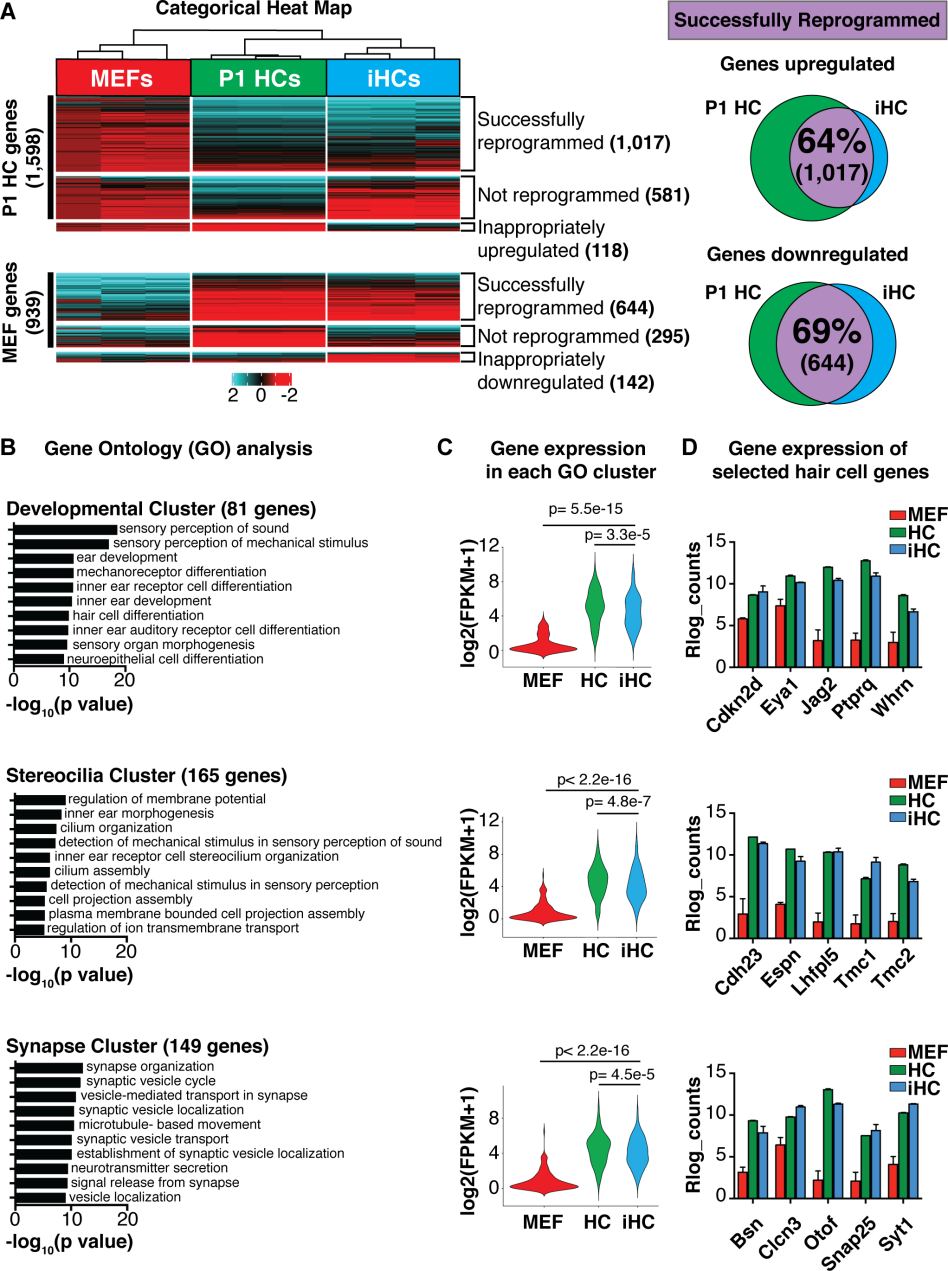
Transcriptional analysis of induced hair cells (iHCs) indicates expression profile similarity to primary cochlear hair cells. (**A**) Categorical heat-map comparing gene expression (RNA-seq) of mouse embryonic fibroblasts (MEFs), P1 cochlear hair cells (P1 HCs), and induced hair cells (iHCs). (n = 3 replicates for P1 HCs, n = 3 replicates for iHCs, n = 3 replicates for MEFs). Venn diagrams show percent of correctly reprogrammed genes. 64% of uniquely expressed P1 HC genes are correctly upregulated during reprogramming, and 69% of inappropriately expressed MEF genes are downregulated. (**B**) Gene Ontology analysis (GO-terms) of successfully upregulated genes in iHCs categorized into three relevant gene clusters: Development, Stereocilia and Synapse. (**C**) RNA expression of the gene set associated with each GO Cluster. Violin plots show relative expression of genes in each of the three clusters: Development (81 genes), Stereocilia (165 genes) and Synapse (149 genes). Gene lists in Figure 2—figure supplement 1. (**D**) Gene expression (Rlog counts) in MEFs, iHCs and P1 HCs for selected hair cell-enriched genes in each of the three clusters. All genes are significantly upregulated in iHCs compared to MEFs with p<0.05 and FDR < 0.01. (n = 3 replicates for P1 HCs, n = 3 replicates for iHCs, n = 3 replicates for MEFs; mean ± SEM). Figure 2—source data 1.Gene Ontology gene lists Gene Ontology (GO) analysis showed that the genes successfully upregulated during reprogramming were revealed as three clusters of GO terms: development-related GO terms, stereocilia-related GO terms, and synapse-related GO terms.These three clusters of GO terms were used to generate cluster-specific gene sets driving the GO designation. Development GO terms represented 81 genes. Stereocilia GO terms represented 165 genes. Synapse GO terms represented 149 genes. These gene lists were used to calculate the level of gene expression of each GO cluster in MEFs, P1 HCs and iHCs (violin plots, Figure 2C).

Direct lineage reprogramming relies on the forced expression of transcription factors to induce a molecular rewiring of the transcriptional programs that characterize specialized cells (Takahashi and Yamanaka, 2006; Takahashi et al., 2007). This involves both upregulation of the target cell-specific genes, in this case of primary cochlear hair cells, and downregulation of the starting cell-specific genes, in this case of MEFs. To assay the extent to which induced hair cells replicate the mouse primary cochlear hair cell gene expression program, we performed RNA-sequencing on FACS-purified *Atoh1*-nGFP+ cells generated by overexpression of *Six1*, *Atoh1*, *Pou4f3*, and *Gfi1* (SAPG) at 14 days post infection (dpi)(hereafter referred to as iHCs). We compared the gene expression of the iHCs to FACS-purified *Atoh1*-nGFP+ primary cochlear hair cells at postnatal day 1 (P1; hereafter referred to as P1 HCs), and MEFs infected with a control retrovirus expressing a fluorescent protein (dsRed; hereafter referred to as MEFs). We categorized the gene expression in iHCs as either ‘successfully reprogrammed’, ‘not-reprogrammed’ or ‘inappropriately expressed’ and divided the categories into those genes that are normally expressed in P1 HCs, but not in MEFs (P1 HC genes, black bar), and those that are normally expressed in MEFs, but not in primary hair cells (MEF genes, black bar)(Figure 2A). From this analysis we determined that the iHCs had transcriptionally activated a hair cell-like signature by successfully upregulating 64% of P1 HC genes, while simultaneously becoming distinct from the starting MEF population by successfully downregulating 69% of MEF genes (Figure 2A). These percentages are comparable to those achieved in the TF-induced direct conversion of MEFs into spinal motor neurons, as well as those attained in MEF-to-cardiomyocyte and hepatocyte-to-neuron direct conversion (Ieda et al., 2010; Marro et al., 2011; Gopalakrishnan et al., 2017; Ichida et al., 2018). These results suggest that iHCs largely resemble primary P1 cochlear hair cells at the transcriptional level.

**Figure 2—figure supplement 1.**
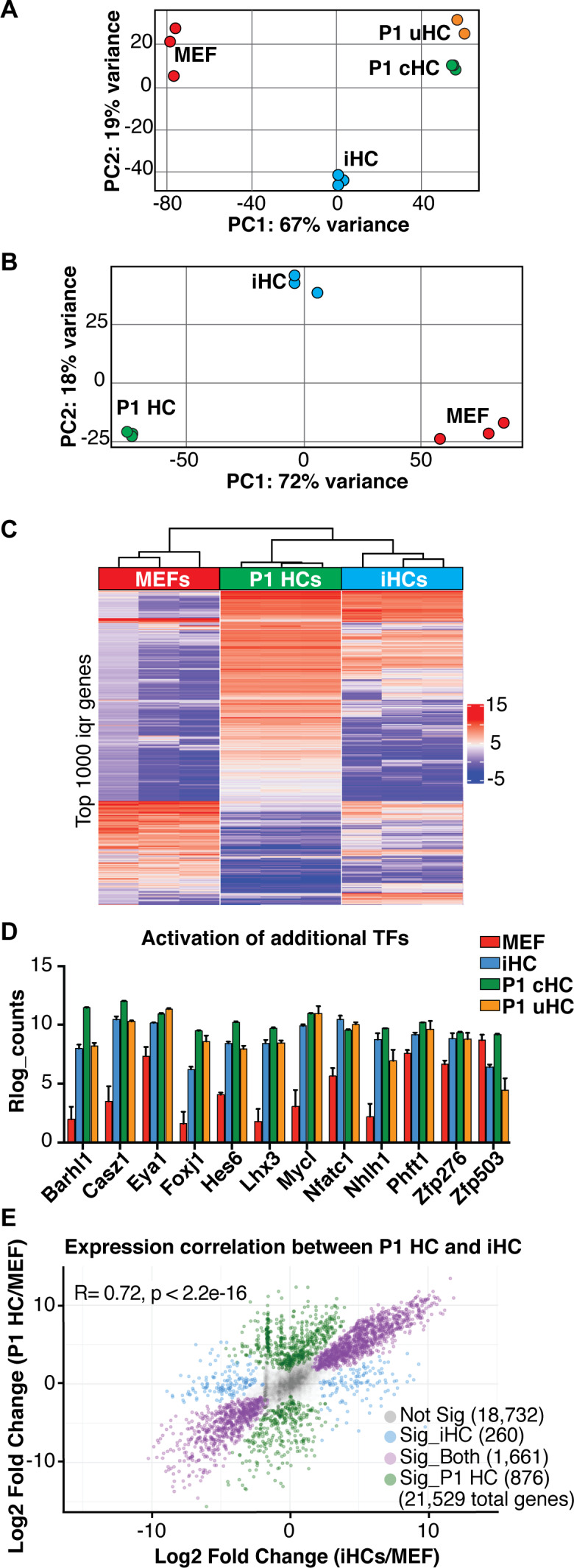
Transcriptional analysis of induced hair cells (iHCs) indicates expression profile similarity to primary cochlear hair cells. (**A**) Principle component analysis (PCA) shows RNA expression profiles for MEFs, P1 cochlear hair cells (P1 cHCs), P1 utricular hair cells (P1 uHCs), and induced hair cells (iHCs). (n = 3 replicates for MEFs, n = 3 replicates for P1 cHCs, n = 2 replicates for P1 uHCs, n = 3 replicates for iHCs). (**B**) Principle component analysis (PCA) shows RNA expression profiles for MEFs, P1 cochlear hair cells (P1 HCs), and induced hair cells (iHCs). (n = 3 replicates for MEFs, n = 3 replicates for P1 cHCs, n = 3 replicates for iHCs). (**C**) Heat map of unsupervised hierarchical clustering shows the consistency between replicates across bulk RNA sequencing profiles for P1 cochlear hair cells (P1 HCs), induced hair cells (iHCs), and MEFs. (n = 3 replicates for P1 HCs, n = 3 replicates for iHCs, n = 3 replicates for MEFs). (**D**) Gene Expression (Rlog counts) in MEFs, iHCs, P1 cHCs and P1 uHCs for the additional 12 transcription factors (TFs) used in the initial set of 16 TFs identified for reprogramming. iHCs are able to activate expression of all TFs with the exception of Zfp503. (n = 3 replicates for MEFs, n = 3 replicates for P1 cHCs, n = 2 replicates for P1 uHCs, n = 3 replicates for iHCs; mean ± SEM). (**E**) Scatter plot of the differential gene expression levels in P1 hair cells (P1 HCs) and induced hair cells (iHCs). Both expression profiles were normalized to the MEF gene expression profile. Expression profiles of P1 HCs and iHCs show strong correlation (R = 0.72, p<2.2e-16).

Nonetheless, a number of genes did not respond to the SAPG group of transcription factors used for reprogramming. Of the 1506 genes expressed in P1 HCs, but not MEFs, 36% were not successfully upregulated in the iHCs, and 118 genes were inappropriately upregulated. Of the 939 genes that are expressed in MEFs, but not in P1 HCs, and thus need to be downregulated during reprogramming, 31% failed to downregulate, and 142 genes were inappropriately downregulated. PCA analysis of bulk RNA-seq data from MEFs, compared to either primary P1 HCs or iHCs show the relative difference between these populations (Figure 2—figure supplement 1B).

Gene Ontology (GO) analysis showed that the genes successfully upregulated during reprogramming were significantly enriched for ‘sensory perception of sound’ and ‘detection of mechanical stimulus’, which revealed as three clusters of genes (Figure 2B). The first cluster was enriched for development-related GO terms such as ‘inner ear receptor cell development’, ‘mechanoreceptor differentiation’, and ‘hair cell differentiation’ (Figure 2B). The second cluster was enriched for stereocilia-related GO terms such as ‘plasma membrane bound cell projection assembly’, ‘cilium organization’ and ‘cilium movement’ (Figure 2B). The third cluster was enriched for synaptic signaling GO terms such as ‘establishment of synaptic vesicle localization’, ‘synaptic vesicle cycle’ and ‘neurotransmitter secretion’ (Figure 2B). These three clusters of GO terms were used to generate cluster-specific gene sets driving the GO designation (Figure 2C, Figure 2—source data 1). Expression levels of each GO cluster-specific gene set, in each cell type, were plotted to visualize the statistically significant iHC divergence from MEFs, and iHC convergence towards a P1 HC expression profile (Figure 2C). This analysis also revealed a significant difference in the level of gene expression (p<0.05) between iHCs and P1 HCs (Figure 2C). This difference may be explained by the maturity level of the iHCs as well as the presence of the residual MEF transcriptional profile. However, further investigation of the Rlog count values for several key genes in each GO cluster demonstrated that the iHCs efficiently upregulated important hair cell genes including *Whirlin* (*Whrn*) involved in cell polarity, *Cadherin23* (*Cdh23*) and *Espin* (*Espn*) important for stereocilia organization and functionality, as well as *Bassoon* (*Bsn*) and *Otoferlin* (*Otof*) required for synaptic scaffolding and synaptic vesicle signaling (Figure 2D). After looking at the expression of key hair cell genes, we determined that the iHCs had also activated all of the initial transcription factors included in the set of 16 candidate factors, with the exception of *Zfp503* (Figure 2—figure supplement 1D). Together, the RNA sequencing results indicate that the iHCs generated by *Six1, Atoh1, Pou4f3*, and *Gfi1* (SAPG) overexpression are capable of repressing most of the initial MEF gene signature, while simultaneously adopting a gene expression signature similar to primary P1 HCs.

Interestingly, the reprogramming transcription factors (SAPG), are normally expressed in both cochlear and utricular differentiating hair cells. We compared postnatal day one cochlear hair cells (P1 cHCs) and utricular hair cells (P1 uHCs) to iHCs. PCA analysis shows that iHCs have not become more similar to one of the two primary hair cell populations (Figure 2—figure supplement 1A). The PCA also demonstrates that at this early developmental time point the P1 cHCs and P1 uHCs are themselves immature, and have similar transcriptional profiles. Nonetheless, the expression profile of iHCs has drastically shifted away from the MEF signature and towards an immature hair cell-like signature.

### Induced hair cells are distinct from other *Atoh1* dependent lineages

**Figure 3.**
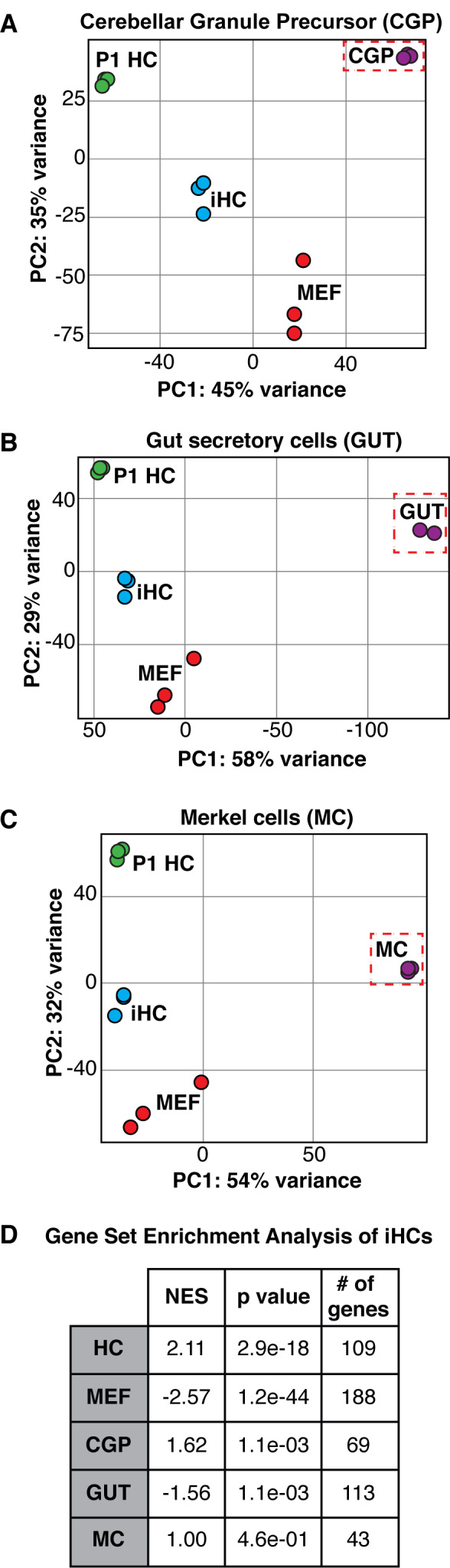
The expression profile of induced hair cells resembles primary hair cells and is distinct from other *Atoh1-*dependent lineages. (**A–C**) Principle component analysis showing the differences in transcriptional profiles between P1 cochlear hair cells (P1 HCs), induced hair cells (iHCs), control mouse embryonic fibroblasts (MEFs), and cerebellar granule precursor cells (CGP), secretory cells of the gut (GUT), and Merkel cells (MC), respectively. (**D**) Gene Set Enrichment Analysis (GSEA) comparing iHC expression profile to the unique gene sets of MEFs, HCs, CGPs, GUT cells, and MCs. Table reports normalized enrichment score (NES), p values, and number of genes in each gene set. Normalized Enrichment Score (NES) shows the strongest correlation between gene sets was between iHCs and the primary hair cell (HC) gene set. Gene signature lists in Figure 3—source data 1. Figure 3—source data 1.Gene Set Enrichment Analysis gene lists Gene Set Enrichment Analysis (GSEA) (Subramanian et al., 2005) was used to compare the transcriptomes in MEFs, P1 hair cells (HC), P1 Cerebellar granule precursors (CGP), adult Gut secretory cells (GUT), and P1 Merkel cells (MC).We defined groups of genes as part of a specific signature for each cell type. MEF signature represents 188 genes. HC signature represents 109 genes. MC signature represents 43 genes. CGP signature represents 69 genes. GUT signature represents 113 genes. These gene signatures were used to calculate Normalized Enrichment Scores (NES) (Subramanian et al., 2005) and p-values for each cell type in comparison to iHCs (table, Figure 3D).

Expression of *Atoh1* is necessary and sufficient for hair cell differentiation in the context of the inner ear primordium (Bermingham et al., 1999; Chen et al., 2002; Woods et al., 2004; Chonko et al., 2013; Cai et al., 2013), however several other lineages including cerebellar granule cell progenitors (Klisch et al., 2011), Merkel cells (Ostrowski et al., 2015), and the secretory cell lineage of the gut (Kim et al., 2014) rely on *Atoh1* expression for differentiation. To characterize the specificity of our reprogramming to the hair cell-like state, we analyzed RNA sequencing data from FACS-purified iHCs relative to other *Atoh1*-dependent lineages including cerebellar granule precursors (CGP), secretory cells of the gut (GUT), and Merkel cells (MC). Principle component analysis (PCA) indicated that iHCs were more similar to primary P1 cochlear hair cells (P1 HC) than to either cerebellar granule cell precursors (CGP) (Figure 3A), secretory cells of the gut (GUT) (Figure 3B), or Merkel cells (MC) (Figure 3C), showing that they established a hair cell-specific transcriptional program and have not adopted the transcriptional profile of other *Atoh1*-dependent lineages.

As an additional test, we compared iHCs to the other *Atoh1*-dependent lineages using Gene Set Enrichment Analysis (GSEA) (Subramanian et al., 2005). By comparing the transcriptomes in MEFs, P1 hair cells (HC), P1 Cerebellar granule precursors (CGP), adult gut secretory cells (GUT), and P1 Merkel cells (MC), we defined groups of genes as part of a specific signature for each cell type (Figure 3—source data 1). The GSEA program identified gene-lists exclusive to each cell type; these gene-lists included only genes which were not expressed in any two cell types. We calculated Normalized Enrichment Scores (NES) (Subramanian et al., 2005) for each cell type in comparison to iHCs. The largest NES was for the comparison of iHCs to P1 hair cells (HC), indicating an enrichment for the HC gene signature, while showing lower enrichment scores, and even negative enrichment scores, for the other cell type comparisons (Figure 3D). Thus, reprogramming with SAPG establishes a hair cell-like transcriptional program without adopting the transcriptional profiles of other *Atoh1*-dependent lineages.

### Chromatin accessibility profile of induced hair cells resembles that of primary cochlear hair cells

**Figure 4.**
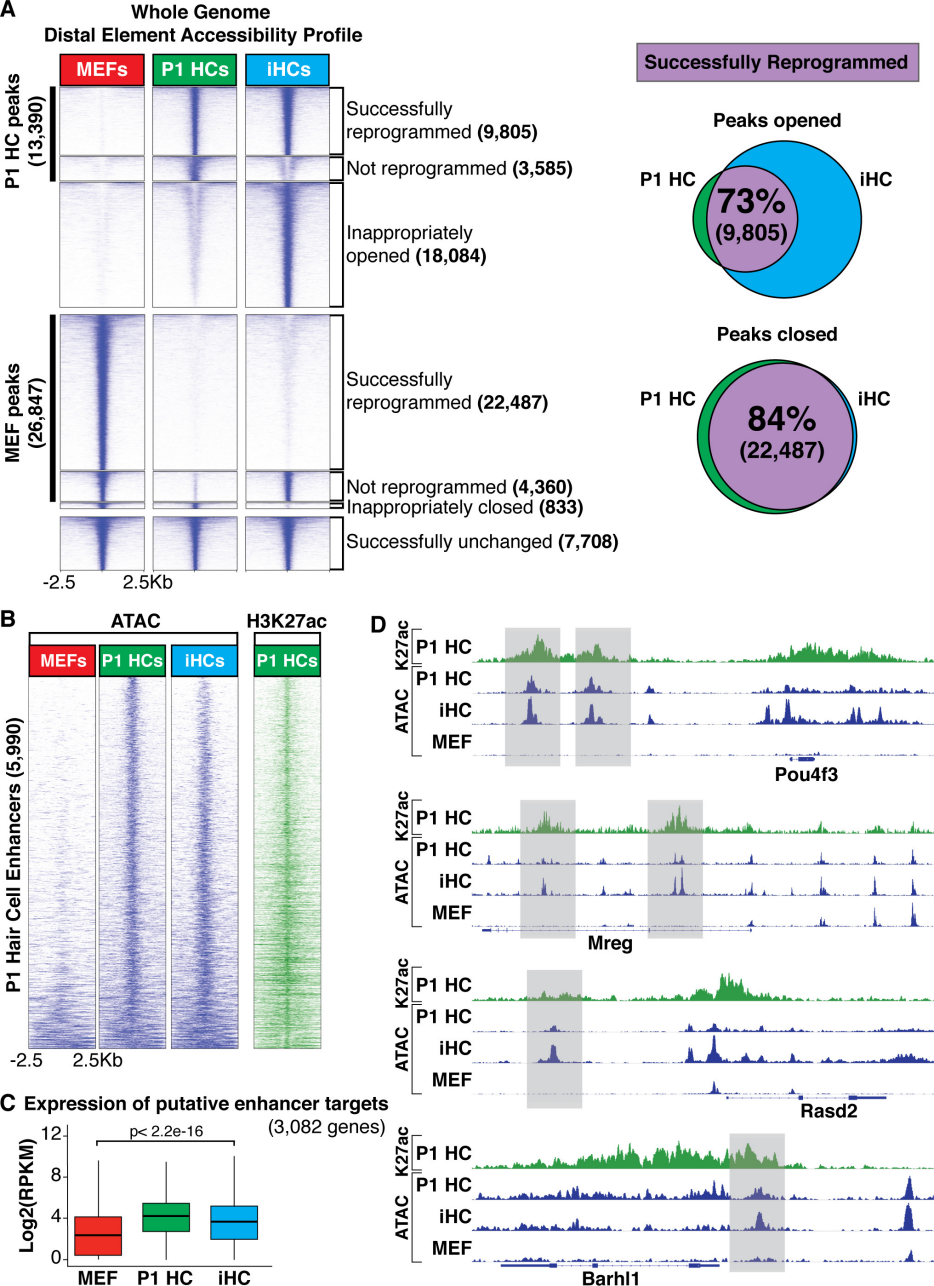
Chromatin accessibility of induced hair cells (iHCs) indicates profile similarity to primary cochlear hair cells. (**A**) Heat map comparing genome wide chromatin accessibility profiles of MEFs, P1 HCs, and iHCs. Accessibility is divided into seven clusters: 1) successfully reprogrammed HC peaks, 2) not reprogrammed HC peaks, 3) inappropriately opened peaks, 4) successfully closed MEF peaks, 5) not reprogrammed MEF peaks, 6) inappropriately closed peaks, and 7) successfully unchanged peaks. Scale of each sample column is ± 2.5 Kb from ATAC peak. Venn diagrams show percent of correctly reprogrammed chromatin regions. 73% of unique P1 HC chromatin regions are successfully opened during reprogramming, and 84% of MEF chromatin regions are successfully closed in reprogramming. (**B**) Heat map comparing chromatin accessibility at primary cochlear hair cell enhancers. Enhancers were identified as regions with open chromatin ATAC peaks and H3K27ac in P1 HCs. ChIP data for H3K27ac shown as green heat map and ATAC chromatin accessibility data of the respective regions shown as blue heat maps. Heat maps ordered from low to high information content. iHCs successfully open P1 HC enhancer regions that were closed in the starting MEF population. Scale of each sample column is ± 2.5 Kb from ATAC peak. (**C**) Expression levels (Log2(RPKM)) of 3082 putative primary hair cell enhancer targets. Enhancer targets were identified by mapping to the nearest transcription start site for each enhancer and the expression of each putative target was acquired from the RNA-seq results. iHCs significantly upregulate the expression of the putative primary hair cell enhancer targets. (**D**) Integrative Genomics Viewer (Robinson et al., 2011) tracks show primary hair cell H3K27ac profile alongside chromatin accessibility profiles of P1 HCs, iHCs and MEFs. Chromatin accessibility changes at specific hair cell enhancers for *Pou4f3, Mreg, Rasd2* and *Barhl1* are highlighted in grey boxes.

Chromatin structure controls the accessibility of genes for either activation or repression in response to developmental and environmental signaling (Volpi et al., 2000; Kozubek et al., 2002; Goetze et al., 2007; Buenrostro et al., 2015b; Chen et al., 2016; Sijacic et al., 2018), and as such, is an important regulator of cell type-specific gene expression. We used an Assay of Transposase Accessible Chromatin (ATAC) sequencing (Buenrostro et al., 2015a; Chen et al., 2016) to analyze the regions of open/accessible chromatin in MEFs (MEF peaks), primary P1 hair cells (P1 HC peaks), and iHCs (Figure 4A). As in our analysis of gene expression (Figure 2), we characterized the open chromatin regions into those that are present in primary P1 HCs, but not in MEFs (P1 HC peaks, black bar), and those that are open in MEFs, but not primary HCs (MEF peaks, black bar), as analyzed by ATAC-seq accessibility (Figure 4A). We defined these groups, as in Figure 2, as either ‘successfully reprogrammed’, ‘not reprogrammed’, and ‘inappropriately opened/closed’ (i.e. not matching either P1 HC peaks or MEF peaks).

The iHCs show robust opening of de novo distal element regions of the chromatin that are open in P1 HCs, as well as large-scale chromatin closing in regions of the genome that were accessible in the starting MEF population. The hair cell-appropriate changes in chromatin accessibility of iHCs are also accompanied by a proportion of inappropriate opening or closing of chromatin regions. Of the 13,390 peaks present uniquely in P1 HCs, 73% were successfully opened during reprogramming, while 27% were not opened during reprogramming, and an additional 18,084 peaks opened inappropriately in iHCs (Figure 4A). Conversely, of the 26,847 peaks unique to MEFs, 84% of peaks were successfully closed during reprogramming, 16% were not closed during reprogramming, and an additional 833 peaks were inappropriately closed during reprogramming (Figure 4A).

Since most distal accessible elements are not active enhancers in a given cell type (Heintzman et al., 2007), we analyzed the H3K27ac-state of the distal elements present in P1 HCs, a marker of active enhancers (Creyghton et al., 2010; Figure 4B). These results show that most of the enhancers identified in P1 HCs are opened in iHCs. Global enhancer targets have not been analyzed in these cell types due to small numbers, so we arbitrarily assigned putative gene targets to each P1 HC enhancer by identifying the closest transcriptional start site. This is expected to identify 27–47% of genuine targets, based on chromosome conformation capture experiments performed in other cell types (Sanyal et al., 2012). Based on our RNA-seq data, we found that the genes defined as putative targets of P1 HC-specific enhancers had significantly higher expression in both P1 HCs and iHCs compared to MEFs (Figure 4C).

To visualize the ATAC-seq and ChIP-seq data at specific loci we used the Integrative Genomics Viewer (IGV) (Robinson et al., 2011). We chose four known hair cell loci, *Pou4f3, Mreg, Rasd2*, and *Barhl1*, that exemplify the changes in chromatin structure at H3K27ac-defined enhancers between P1 HCs, iHCs and MEFs (Figure 4D). These results indicate that robust and hair cell-appropriate global changes in chromatin accessibility accompany the large shift in the transcriptional profile of iHCs.

### *Six1*, *Atoh1*, *Pou4f3*, and *Gfi1* are capable of reprogramming postnatal and adult somatic cells

**Figure 5.**
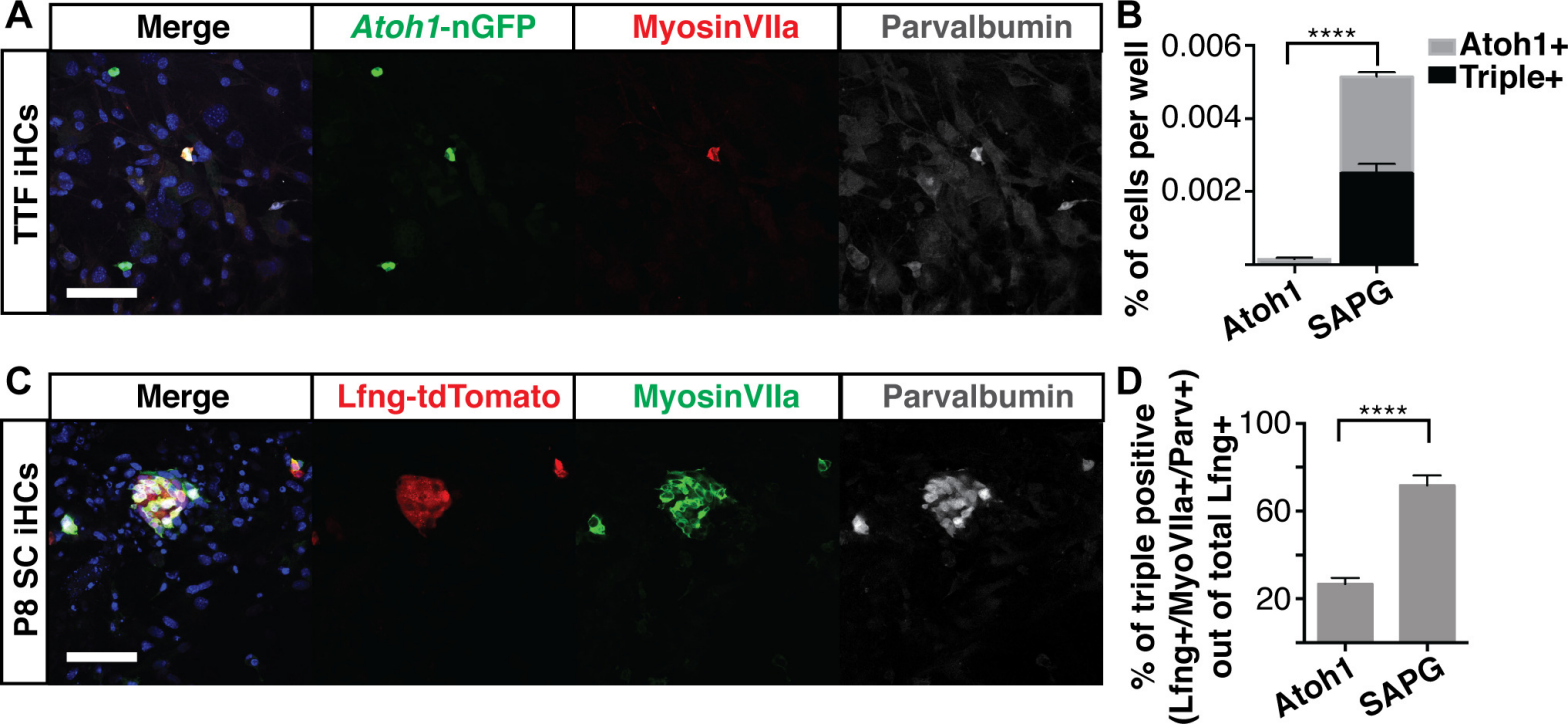
Six1, Atoh1, Pou4f3 and Gfi1 are capable of reprogramming adult cells. (**A**) Adult mouse tail tip fibroblasts (TTFs) were infected with SAPG and allowed to reprogram for 14 days before fixation and immunostaining. Reprogrammed TTF are able to activate the *Atoh1*-nGFP reporter and stain for anti-MyosinVIIa (red) and anti-Parvalbumin (grey). Merged image includes Hoechst nuclear stain (blue). TTFs were reprogrammed in HCM supplemented with 10% FBS and RepSox. Scale bar represents 50 um. (**B**) Quantification of *Atoh1*-nGFP+ cells and triple positive cells (*Atoh1-*nGFP+/MyosinVIIa+/Parvalbumin+) in TTFs infected with *Atoh1* alone or SAPG. TTFs infected with SAPG generate significantly more *Atoh1*-nGFP+ cells and 48.6% (± 12) of the *Atoh1*-nGFP+ cells are triple positive. TTFs were reprogrammed in HCM supplemented with 10% FBS and RepSox. (N = 3 experiments, n = 3 replicates per experiment, mean ± SEM; Statistics shown for the comparison of triple positive cells in each condition; Student’s t-test *p<0.05, **p<0.01, ***p<0.001, ****p<0.0001). (**C**) Dissociated organs of Corti from P8 transgenic mice with lineage traced supporting cells (*Lfng*-tdTomato+ SC) were infected with SAPG. Cells were reprogrammed for 14 days prior to fixation and immunostaining for anti-MyosinVIIa (green) and anti-Parvalbumin (grey). P8 *Lfng*-tdTomato+ SCs infected with SAPG are able to activate primary hair cell markers MyosinVIIa and Parvalbumin. Scale bar represents 50 um. (**D**) Quantification of the percent of triple positive cells (*Lfng*-tdTomato+/MyosinVIIa+/Parvalbumin+) out of the total number of *Lfng*-tdTomato+ supporting cells per well in cultures infected with *Atoh1* alone or SAPG. Presence of the *Lfng*-tdTomato reporter is independent of the viral infection. With *Atoh1* alone 26.8% (± 7) of the total *Lfng*-tdTomato+ supporting cells are able to activate the primary hair cell markers MyosinVIIa and Parvalbumin, while with SAPG 71.8% (± 12) of the total *Lfng*-tdTomato+ supporting cells are able to activate primary hair cell markers. (n = 7 replicates, mean ± SEM; Student’s t-test *p<0.05, **p<0.01, ***p<0.001, ****p<0.0001).

We have used mouse embryonic fibroblasts (MEFs) as a starting cell type for our reprogramming efforts in the experiments described thus far. However, MEFs are an embryonic and relatively heterogeneous cell population (Singhal et al., 2016). To test the ability of the SAPG transcription factors to reprogram a mature somatic cell from a heterologous lineage, we chose as proof-of-principle, to reprogram *Atoh1*-nGFP transgenic adult tail tip fibroblasts (TTFs). We virally transduced TTFs with *Six1*, *Atoh1*, *Pou4f3*, and *Gfi1* (Figure 5A). While TTFs are known to be infected by retrovirus at a lower efficiency than MEFs (Liu et al., 2011; Lalit et al., 2016), SAPG activated the *Atoh1-*nGFP reporter in adult tail tip fibroblasts (Figure 5A). In addition, SAPG catalyzed the expression of MyosinVIIa and Parvalbumin, indicating that as in MEFs, these transcription factors can convert adult tail tip fibroblasts into *Atoh1-*nGFP+/MyosinVIIa+/Parvalbumin+ iHCs (Figure 5A,B).

**Figure 5—figure supplement 1.**
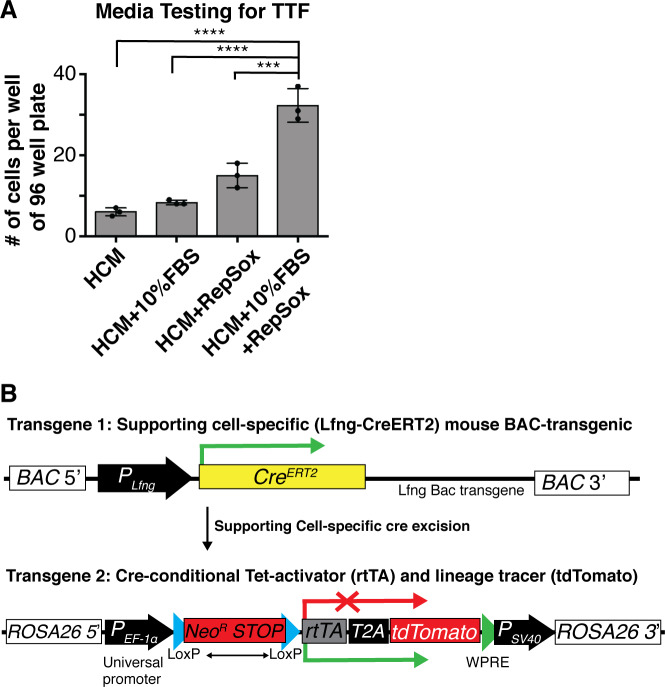
Six1, Atoh1, Pou4f3 and Gfi1 are capable of reprogramming adult cells. (**A**) Adult mouse tail tip fibroblasts (TTFs) were infected with SAPG and allowed to reprogram for 14 days before fixation and quantification. Regular hair cell media (HCM) was not optimal to culture TTF post-infection and caused severe loss of cells. TTF survival and reprogramming was best with the addition on 10% FBS and RepSox to the Hair Cell Media (HCM). (N = 3 experiments, n = 3 replicates per experiment, mean ± SEM; Student’s t-test *p<0.05, **p<0.01, ***p<0.001, ****p<0.0001). (**B**) Schematic of transgenes used for lineage tracing of Lunatic fringe (Lfng) positive supporting cells.

Supporting cells are an attractive target for gene therapy approaches to hair cell regeneration, due to their known role in regeneration in non-mammalian vertebrates (Corwin and Cotanche, 1988; Ryals and Rubel, 1988; Stone and Cotanche, 2007; Brignull et al., 2009), their having a common progenitor with hair cells (Fekete et al., 1998; Kelley, 2006; Driver et al., 2013), and their survival in long-deafened mice (Oesterle and Campbell, 2009). Although hair cells do not regenerate in the mature mammalian cochlea, perinatal supporting cells have been shown to have a transient ability to directly transdifferentiate into hair cells in response to Atoh1 (Kelly et al., 2012; Liu et al., 2012b), or loss of Notch-mediated lateral inhibition (Mizutari et al., 2013; Maass et al., 2015), however this potential is lost at very early postnatal stages (White et al., 2006; Takebayashi et al., 2007; Doetzlhofer et al., 2009; Liu et al., 2012a; Cox et al., 2014; Bramhall et al., 2014).

One plausible route to the in vivo regeneration of hair cells in the organ of Corti would be the conversion of mature supporting cells into hair cells in long deafened individuals. *Atoh1* alone can convert perinatal supporting cells into hair cells (Kelly et al., 2012; Liu et al., 2012b; Yang et al., 2013), but transdifferentiation potential decreases rapidly thereafter, such that by two weeks of age, neither *Atoh1* expression, nor induction of transdifferentiation by Notch-inhibition, can induce the transdifferentiation of supporting cells to a hair cell fate (Maass et al., 2015; Jiang et al., 2016). To determine if *Six1*, *Atoh1*, *Pou4f3*, and *Gfi1* (SAPG) are together able to convert supporting cells into hair cells from organs that had passed this transdifferentiation-permissive stage, we labeled P1 supporting cells using a transgenic cross, Lfng-CreERt2::Rosa26^tdTomato^, which allows for permanent labeling of supporting cells with a tdTomato fluorescent marker (Figure 5—figure supplement 1B). We dissociated organs of Corti from *Lfng*-CreERt2::Rosa26^tdTomato^ mice at P8, a time when induced-transdifferentiation is no longer possible, and transduced them with virus encoding *Atoh1* alone, or the combination of four factors, SAPG. Cells were infected and allowed to reprogram for two weeks before immunostaining for MyosinVIIa and Parvalbumin (Figure 5C). The lineage traced cells, from now on referred to as *Lfng*-tdTomato-positive P8 supporting cells (P8 SC) transduced with the SAPG produced significantly more cells that activated MyosinVIIa and Parvalbumin than *Atoh1*-transduced supporting cells (Figure 5D). Since the *Lfng*-tdTomato reporter is independent of the viral SAPG, the percent of triple positive (*Lfng*-tdTomato+/MyosinVIIa+/Parvalbumin+) iHCs was calculated from the total number of *Lfng*-tdTomato-positive cells per well. These results indicate that the combination of *Six1*, *Atoh1*, *Pou4f3*, and *Gfi1* can convert adult tail tip fibroblasts and P8 supporting cells into induced hair cells at a significantly greater rate than *Atoh1* alone.

### Morphological characterization of induced hair cells

Sensory hair cells have a very distinct morphology. As their name suggests, these specialized cells possess hair-like actin-based apical membrane protrusions, called stereocilia, that contain at their tips the mechanically gated ion channels required for mechanotransduction (Kawashima et al., 2011; Pan et al., 2013; Holt et al., 2014). Development of stereocilia involves the elaboration of a single primary, tubulin-based, cilium, known as the kinocilium, centered on a cuticular plate of F-actin filaments from which the stereocilia arise as elongated bundles of microvilli (Cotanche and Corwin, 1991; Troutt et al., 1994; Leibovici et al., 2005; Wang et al., 2005; Tarchini et al., 2016; McGrath et al., 2017).

**Figure 6.**
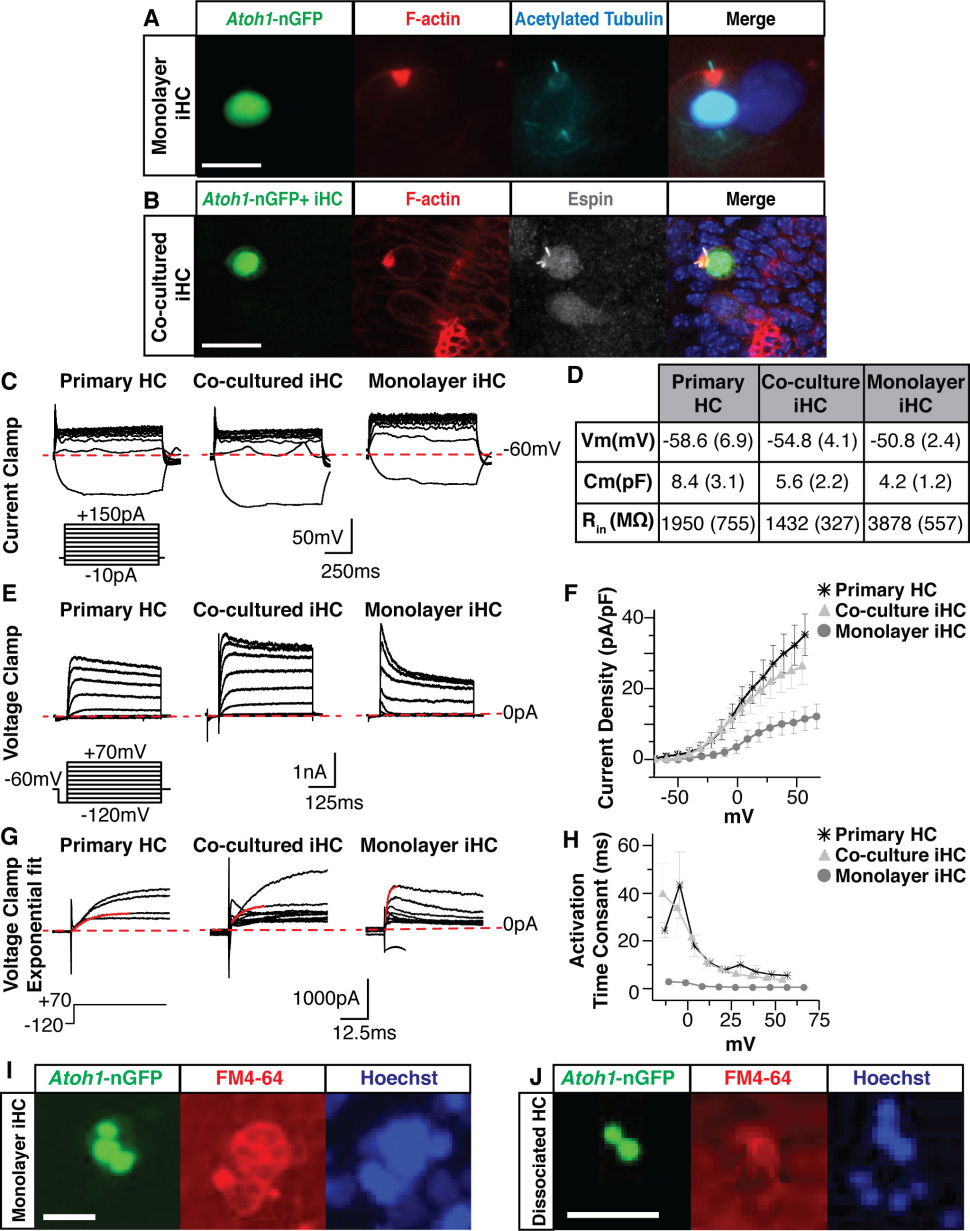
Induced hair cells demonstrate functional properties reminiscent of primary hair cells. (**A**) Images of monolayer-cultured iHCs show polarized F-actin by Phalloidin labeling (red) and a kinocilium by anti-acetylated Tubulin labeling (cyan). Merged image includes Hoechst nuclear stain. Scale bar represents 10 um. (**B**) iHCs co-cultured with dissociated primary E13.5 organs of Corti show an F-actin rich cuticular plate by Phalloidin labeling (red) and stereocilia by anti-Espin labeling (grey). Merged image includes Hoechst nuclear stain. Scale bar represents 20 um. (**C**) Whole cell patch clamping was performed on P1 HCs from a dissociated organ of Corti, co-cultured iHCs and monolayer-cultured iHCs. Results from current clamp show the change in cell voltage as a response to an applied current. Dashed red line represents −60 mV. Current clamp protocol shows steps from −10 to +150 pA in 20 pA increments. Scale bars represent 50 mV on X-axis and 250 ms on Y-axis. (**D**) Basic membrane properties were calculated from the current clamp data to report resting membrane potential (Vm), membrane capacitance (Cm) and input resistance (Rin). Table reports mean (SEM) for each value. (**E**) Results from voltage clamp shows the current output of the cell as a response to applied voltage for primary HCs, co-cultured iHCs and monolayer-cultured iHCs. Dashed red line represents 0 pA. Voltage clamp protocol shows steps from −120 to +70 mV in 10 mV increments. Scale bars represent 1 nA on X-axis and 125 ms on Y-axis. (**F**) IV curve plotting current density (normalized for cell size) as a function of applied voltage for primary HCs, co-cultured iHCs and monolayer-cultured iHCs. Co-cultured iHCs show similar current output to P1 primary hair cells. (**G**) Exponential fits to the voltage clamp traces were used to calculate the current activation time constants for primary HCs, co-cultured iHCs and monolayer-cultured iHCs. Dashed red line represents 0 pA. Solid red line shows exponential fit to outward currents when clamped from −120 mV to +70 mV. Scale bars represent 1000 pA on X-axis and 12.5 ms of Y-axis. (**H**) Current activation time constants reported for P1 HCs, cocultured iHCs and monolayer-cultured iHCs. Co-cultured iHCs show similar current activation kinetics to P1 HCs. (**I**) iHCs expressing the *Atoh1*-nGFP reporter accumulate the styryl dye FM4-64. Image taken after 30 s of incubation with FM4-64. Nuclei labeled in blue using NucBlue live dye. Scale bar represents 25 um. (**J**) Dissociated primary hair cells expressing the *Atoh1*-nGFP reporter accumulate the styryl dye FM4-64. Image taken after 30 s of incubation with FM4-64. Nuclei labeled in blue using NucBlue live dye. Scale bar represents 25 um.

To assess the morphological properties of iHCs we performed immunostaining at 14 days post-infection following SAPG reprogramming, which is approximately 10 days after initial *Atoh1*-nGFP detection. At this time, iHCs exhibited highly polarized F-actin staining as observed by well-defined labeling of Phalloidin-Rhodamine near the apical surface and a primary cilium that labels with antibody to acetylated tubulin, and is centered on the nascent cuticular plate (Figure 6A). This highly polarized pattern is reminiscent of hair cells in both the developing cochlear and vestibular systems (Cotanche and Corwin, 1991; Troutt et al., 1994; Leibovici et al., 2005; Wang et al., 2005; Tarchini et al., 2016; McGrath et al., 2017).

**Figure 6—figure supplement 1.**
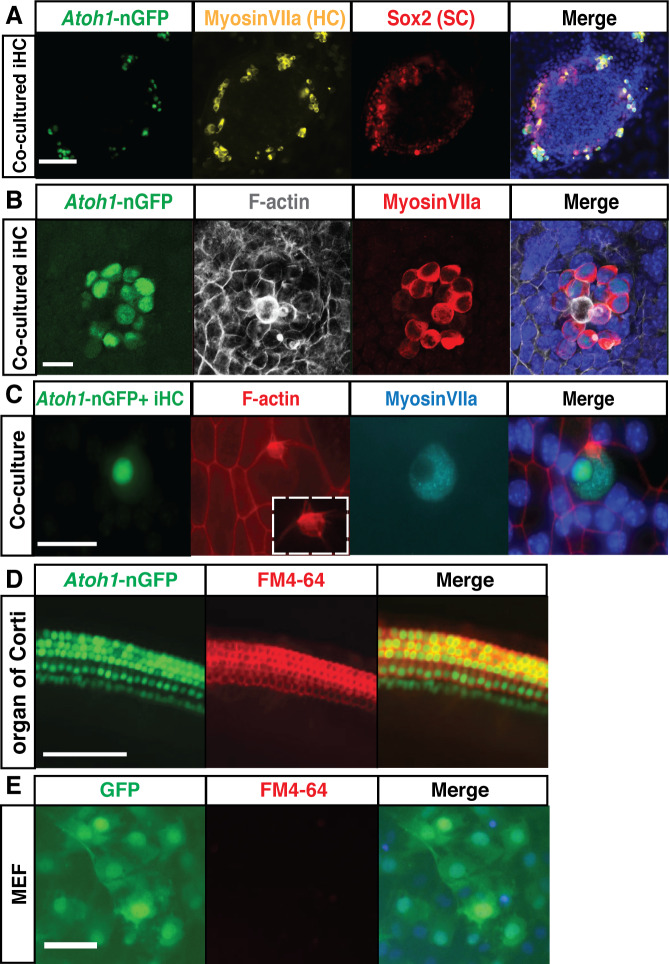
Induced hair cells demonstrate functional properties reminiscent of primary hair cells. (**A**) iHCs (*Atoh1*-nGFP+/MyosinVIIa+) co-cultured with dissociated primary E13.5 organs of Corti organize into epithelial islands alongside primary hair cells (*Atoh1*-nGFP-/MyosinVIIa+) and primary supporting cells (Sox2+). Merged image includes Hoechst nuclear stain. Scale bar represents 100 um. (**B**) iHCs co-cultured with dissociated primary E13.5 organs of Corti show F-actin polarization by Phalloidin labeling (grey) and express MyosinVIIa (red). Merged image includes Hoechst nuclear stain. Scale bar represents 20 um. (**C**) iHCs co-cultured with dissociated primary E13.5 organs of Corti show F-actin rich projections by Phalloidin labeling (red) and express MyosinVIIa (cyan). Scale bar represents 20 um. (**D**) Organ of Corti explants from *Atoh1*-nGFP transgenic mice accumulate the styryl dye FM4-64 specifically in the hair cells. Image taken after 30 s of incubation with FM4-64. Merged image includes Hoechst nuclear stain. Scale bar represents 100 um. (**E**) MEFs infected with eGFP control virus do not accumulate the styryl dye FM4-64. Image taken after 30 s of incubation with FM4-64. Merged image includes Hoechst nuclear stain. Scale bar represents 50 um.

Previous work has demonstrated that mixing dissociated cells from embryonic or perinatal organ of Corti with periotic mesenchyme, allows them to rapidly self-organize and differentiate in vitro into epithelial island-like structures (Doetzlhofer et al., 2004; White et al., 2006). To determine if iHCs are capable of integrating appropriately into these sensory epithelial-like structures which contain both primary hair cells and supporting cells, we FACS-purified *Atoh1*-nGFP+ iHCs and mixed them with dissociated primary embryonic (E13.5) sensory epithelium, containing primary hair cells, primary supporting cells, and a portion of the surrounding periotic mesenchyme (Figure 6—figure supplement 1A-C). After two weeks of co-culture, iHCs contained polarized cuticular plates (F-actin) and stereocilia (espin-positive), and were found incorporated into the epithelial islands containing native hair cells and supporting cells (Figure 6B). Greater than 80% of iHCs that engrafted in epithelial islands exhibited highly polarized F-actin staining (data not shown). Thus, iHCs morphologically resemble primary hair cells and possess properties required for the proper structural integration with primary hair cells and supporting cells.

### Induced hair cells demonstrate voltage-dependent ion currents

To determine if iHCs possess electrophysiological properties similar to those of primary hair cells, we performed whole-cell patch-clamp recordings. We measured the biophysical properties of our cells in voltage-clamp and current-clamp to analyze the voltage-gated currents and passive membrane properties of these cells. We compared primary hair cells (n = 5) with *Atoh1*-nGFP+ iHCs in two experimental conditions: monolayer-cultured iHCs (n = 10) and iHCs co-cultured with dissociated organ of Corti (n = 10). Within co-cultures, the presence of the *Atoh1*-nGFP reporter enabled specific patch clamp analysis of iHCs.

Current-clamp was used to measure the passive membrane properties of primary hair cells, co-cultured iHCs and monolayer-cultured iHCs (Figure 6C). The properties measured included the resting potential, membrane capacitance, and input resistance (Figure 6D). As a negative control we patch-clamped mouse embryonic fibroblasts. The MEFs showed gross electrophysiological properties that did not overlap with that of primary hair cells or induced hair cells (data not shown). The mean resting potentials for primary hair cells, cocultured iHCs, and monolayer-cultured iHCs were −58.6 mV (± 6.9), –54.8 mV (± 4.1), and −50.8 mV (± 2.4), respectively (Figure 6D). These values are comparable to previously reported primary hair cell resting potentials (Dallos, 1985; Oliver et al., 2003). The input resistances were measured to infer the total ion channel composition of the cell. Higher input resistance values indicate the cell may have fewer ion channels to allow current to flow in and out of the plasma membrane. The input resistance was highest in monolayer-cultured iHCs (3878 ± 557 MΩ). However, the input resistance of co-cultured iHCs (1432 ± 327 MΩ) was comparable to that of primary hair cells (1950 ± 755 MΩ) (Figure 6D). Lastly, the capacitance, which can be used to infer the surface area of the cell, was highest in primary hair cells (8.4 ± 3.1 pF), followed by co-cultured iHCs (5.6 ± 2.2 pF) and then monolayer-cultured iHCs (4.2 ± 1.2 pF) (Figure 6D).

In addition, we performed voltage-clamp to measure the magnitude and time dependent activity of the whole-cell currents in primary hair cells, co-cultured iHCs and monolayer-cultured iHCs (Figure 6E). In response to the applied voltage, both primary hair cells and iHCs produced positive-outward currents (Figure 6E). However, the monolayer-cultured iHCs produced relatively small whole-cell currents that rapidly inactivated (Figure 6E). In contrast, primary hair cells and co-cultured iHCs displayed robust outward currents that more slowly inactivated over the course of the protocol (Figure 6E). We measured the steady-state outward current as a function of the voltage-clamp potential and normalized the current magnitude by the cell’s capacitance to analyze current densities. Monolayer-cultured iHCs showed small current densities while the co-cultured iHCs and primary hair cells displayed overlapping magnitudes of voltage-dependent current densities (Figure 6F).

A prominent voltage-clamp feature in primary hair cells is a delayed onset of a slow-activating outward current (Housley and Ashmore, 1992; Marcotti and Kros, 1999). In order to measure the kinetic properties of this slow-activating outward current, we fit a single exponential at the onset of the current (Figure 6G) to compare the mean time constants when the cells were clamped from −120 mV to 70 mV (Figure 6H). The delayed onset current of monolayer-cultured iHCs displayed fast time constants (Figure 6H). In contrast, the co-cultured iHCs and primary hair cells showed similarly longer time constants, indicating that their outward currents have similar activation kinetics (Figure 6H). Together, these electrophysiological data suggest that both monolayer and co-cultured iHCs possess voltage dependent currents, however, when iHCs are co-cultured with dissociated organ of Corti, their size, passive membrane properties and ion channel function are more similar to those of primary hair cells.

### Induced hair cells possess rudimentary mechanotransduction properties

Primary sensory hair cells acquire distinct functional properties early in development in order to properly convert mechanical sound waves into neurotransmitter signaling (Wu et al., 2017). Mechanotransduction relies on the organization of stereocilia, the assembly of tip links, and insertion of mechanically gated ion channels at the tip of each stereocilia (Kawashima et al., 2011; Pan et al., 2013). Mechanotransduction channels are highly permeable to styryl dyes, and their accumulation in hair cells occurs with much faster kinetics than most other cells (Gale et al., 2001).

Primary hair cells within the intact organ of Corti rapidly and selectively accumulate the styryl dye FM4-64 within seconds, a time frame consistent with entry of the dye through mechanotransduction channels rather than endocytosis (Lelli et al., 2009; Figure 6—figure supplement 1D). In contrast, MEFs failed to incorporate FM4-64 within the 30 s time frame (Figure 6—figure supplement 1E). However, iHCs rapidly incorporated FM4-64 to high levels within a 30 s time course (Figure 6I) demonstrating that iHCs possess rudimentary mechanotransduction channels with similar styryl dye uptake as in primary hair cells within the intact organ of Corti (Figure 6—figure supplement 1D) and primary hair cells from dissociated organ of Corti preparations (Figure 6J).

### Induced hair cells recapitulate sensitivity to gentamicin, a known ototoxin

Environmental and pharmacological ototoxins that cause selective degeneration of hair cells are major contributors to hearing loss worldwide (Al-Malky et al., 2015; Sagwa et al., 2015; Knight et al., 2017). Gentamicin is representative of a large class of highly effective aminoglycoside antibiotics that result in significant hair cell degeneration (Alharazneh et al., 2011). Unfortunately, a lack of mammalian models suitable for large scale screening of ototoxins and otoprotectants has restricted the development of small molecules to reduce ototoxicity and identification of compounds that can protect against known ototoxins.

**Figure 7.**
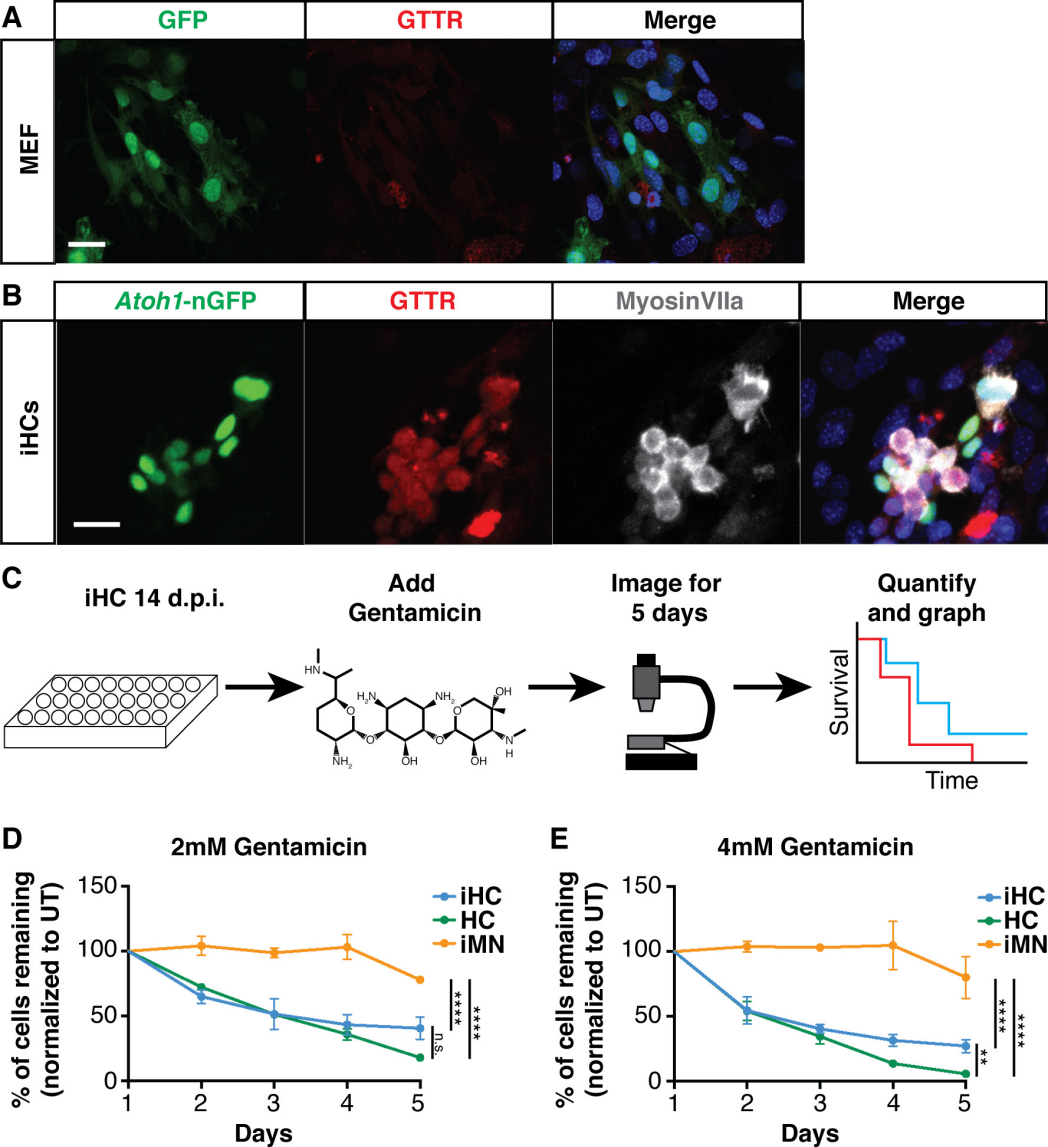
Induced hair cells recapitulate susceptibility to a known ototoxin, Gentamicin. (**A**) MEFs infected with eGFP control virus do not accumulate Gentamicin-Texas Red (GTTR). MEFs were treated with 0.5 mM GTTR for 3 hr. Merged image includes Hoechst nuclear stain. Scale bar represents 50 um. (**B**) iHCs can accumulate GTTR. iHCs were treated with 0.5 mM GTTR for 3 hr. iHCs were also labeled for anti-MyosinVIIa (grey). Merged image includes Hoechst nuclear stain. Scale bar represents 50 um. (**C**) Schematic of experimental design for longitudinal survival of *Atoh1*-nGFP+ iHCs. (**D–E**). Longitudinal survival tracking of P1 hair cells (HC) from dissociated organ of Corti preparations, induced hair cells (iHCs) and induced motor neurons (iMNs) treated with gentamicin at 2 mM and 4 mM respectively. (n = 3 replicates each; mean ± SEM; Two-Way ANOVA *p<0.05, **p<0.01, ***p<0.001, ****p<0.0001).

To determine if iHCs are sensitive to ototoxic compounds, we tested their ability to accumulate gentamicin in a similar manner to primary hair cells. Primary hair cells of the organ of Corti specifically accumulated Texas-Red conjugated gentamicin (GTTR), but not Texas Red (TR) alone when treated with 0.5 mM of either compound for 3 hr (Figure 7—figure supplement 1A, B). MEFs transduced with a GFP-expressing control virus did not accumulate GTTR (Figure 7A). The iHCs, similarly to primary hair cells, robustly accumulated gentamicin-Texas Red (GTTR) (Figure 7B), but not Texas Red (TR) alone (Figure 7—figure supplement 1C), after a 3 hr treatment at 0.5 mM.

**Figure 7—figure supplement 1.**
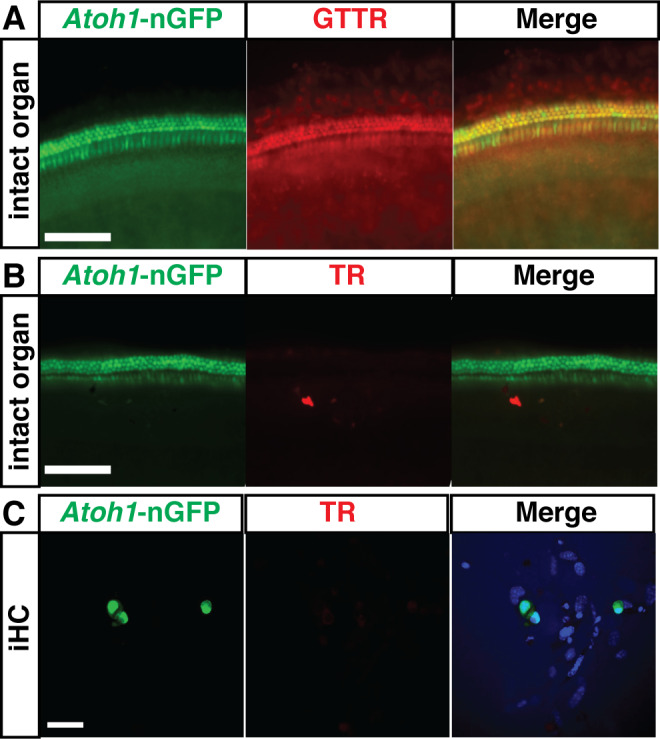
Induced hair cells recapitulate susceptibility to a known ototoxin, Gentamicin. (**A**) Organ of Corti explants from *Atoh1*-nGFP transgenic mice accumulate Gentamicin-Texas Red (GTTR) specifically in the hair cells. Organs were treated with 0.5 mM GTTR for 3 hr. Scale bar represents 100 um. (**B**) Organ of Corti explants from *Atoh1*-nGFP transgenic mice do not accumulate Texas Red (TR) alone. Organs were treated with 0.5 mM TR for 3 hr. Scale bar represents 100 um. (**C**) Induced hair cells (iHCs) do not accumulate Texas Red alone. iHCs were treated with 0.5 mM TR for 3 hr. Merged image includes Hoechst nuclear stain. Scale bar represents 50 um.

To assess whether the gentamicin accumulation seen by GTTR treatment could cause iHCs to degenerate, we established a longitudinal survival assay using robotic imaging and automated tracking of iHC survival (Figure 7C). To selectively identify and track survival of *Atoh1*-nGFP+ iHCs from daily whole-well images, we customized a time-lapse nuclei count recipe running on SVCell RS 4.0 (a product of DRVision Technologies that has been rebranded to Aivia). The software can automatically detect and count iHCs based on nuclei morphology and the *Atoh1*-nGFP fluorescence, with comparable results to manual counting (p=0.53). We performed the survival assay with iHCs, dissociated primary P1 cochlear hair cells (HC) as a positive control, and induced motor neurons (iMNs) as a negative control. While gentamicin caused primary hair cell degeneration in a dose-dependent manner, it caused little-to-no toxicity to *Hb9-*RFP+ induced motor neurons generated from MEFs by transduction with Ngn2, Isl1, Lhx3, Ascl1, Brn2, and Mty1l (Son et al., 2011; Figure 7D,E). Similar to primary hair cells, iHCs treated with gentamicin showed rapid, dose-dependent degeneration (Figure 7D,E). These data indicate that iHCs possess functional properties of primary hair cells and display selective vulnerability to the known ototoxin, gentamicin. Moreover, these results suggest that iHCs provide a scalable platform for detecting agents that protect against gentamicin ototoxicity.

## Discussion

The discovery of treatments for hearing loss, as well as screening for drugs that can protect the sensory hair cells of the inner ear from environmental stress, such as chemotherapy, have been hampered by the small number and inaccessibility of the sensory cells of the inner ear. The current study is an effort to alleviate this problem through the use of direct lineage reprogramming of somatic cells to generate the large numbers of induced hair cells needed for high-throughput screening. To this end, we have succeeded in identifying a cocktail of transcription factors, *Six1, Atoh1, Pou4f3*, and *Gfi1* (SAPG), that is able to reprogram somatic cells to a sensory hair cell-like fate using alternately, mouse embryonic fibroblasts, adult tail tip fibroblasts, and postnatal day eight supporting cells. The reprogramming efficiency using this SAPG cocktail (35%) is similar and in some cases higher than the best reprogramming strategies for other cell types such as cardiomyocytes with 20% efficiency (Ieda et al., 2010) and motor neurons with 10–40% efficiency (Ichida et al., 2009; Babos et al., 2019). With this method, we show that the reprogrammed hair cells are transcriptionally and epigenetically similar to perinatal primary cochlear hair cells, including morphology, physiology, and susceptibility to ototoxic agents, specifically gentamicin. We also demonstrate a method for high-throughput screening, that in the future will allow the discovery of new otoprotectants, as well as gene-therapy/regenerative medicine approaches to treat hearing loss.

### Reprogramming efficiency and the level of maturity of iHCs

As confirmation of the importance of the four transcription factors identified in our unbiased reprogramming investigation, three of the four transcription factors, *Atoh1, Pou4f3*, and *Gfi1*, were previously used to induce a hair cell like-fate from mouse ES cells that had been partially differentiated into ectodermal organoids (Costa et al., 2015). In our hands, these factors are able to activate reporter expression in MEFs from *Atoh1-*nGFP mice, but yield a mixed population in which many GFP-positive cells failed to upregulate the hair cell markers MyosinVIIa and Parvalbumin. We hypothesize that the addition of *Six1* increases reprogramming efficiency by pushing cells towards the sensory ectodermal lineage. *Six1* has been reported previously to promote competency and progenitor-like state, as well as being an essential determinant of early sensory inner ear lineage (Zheng et al., 2003; Ozaki et al., 2004; Zhang et al., 2017). The expression gradient of *Six1* in the developing sensory epithelium precedes the activation of *Atoh1* (Ahmed et al., 2012). *Six1* directly targets the *Atoh1* autoregulatory enhancer, as well as other essential hair cell enhancers for *Pou4f3* and *Gfi1*, with an increase in *Six1* occupancy as hair cells differentiate with in the sensory epithelium (Li et al., 2020). Additionally, *Six1* has been shown to play a role in the maturation of hair cells by regulating key genes involved in the establishment of planar cell polarity and hair-bundle orientation (Li et al., 2020). These studies support our findings that, in addition to *Atoh1, Pou4f3* and *Gfi1*, *Six1* is an important upstream transcription factor in establishing a hair cell-specific gene expression program in our direct reprogramming.

At perinatal times, only modest differences in gene expression are able to distinguish inner and outer hair cells of the cochlea (Liu et al., 2014b), as well as vestibular vs. cochlear hair cells (Burns et al., 2015). The transcriptional profiles of postnatal day one cochlear hair cells and utricular hair cells are very similar (Figure 2—figure supplement 1A). This time point represents an immature hair cell, and the transcriptional profiles are known to change as the hair cells mature and acquire subtype specificity and functionality (Burns et al., 2015; Zhu et al., 2019; Hoa et al., 2020). As a result of comparing iHCs to postnatal day one primary hair cells, we are unable to statistically classify iHCs as being more similar to one or another hair cell type based on the bulk RNA sequencing. While many of the specialized gene characteristics of the cochlear hair cells are clearly upregulated during reprogramming (Figure 2), the iHCs fail to activate other important genes essential for the functional maturation of the sensory receptors in the cochlea, such as *Prestin* and *Gata3* (Liberman et al., 2002; Bardhan et al., 2019).

The imperfections of iHC reprogramming could have several causes. First, as noted, we could be lacking additional, hair cell-type transcription factors to drive cells to a more mature state. For example, the transcription factor Zfp503 identified in the initial set of 16 factors for reprogramming, does not get activated in response to the SAPG reprogramming cocktail. We saw that the addition of Zfp503 to the core group SAPG negatively impacted the efficiency of activation of the *Atoh1*-nGFP reporter (Figure 1—figure supplement 1G), however it remains to be investigated whether the addition of Zfp503 can confer a more mature induced hair cell phenotype. Zfp503 is highly expressed in postnatal day one cochlear hair cells, but not in utricular hair cells, suggesting it could play an important role in conferring subtype specificity in the iHC reprogramming. Additional bioinformatic analysis has shown that some of the relatively small group of distal regulatory elements that are present in P1 hair cells, but fail to open in iHCs, associate with hair cell-specific genes that also fail to be robustly expressed during reprogramming (data not shown). This further suggests additional factors, perhaps such as Gata3, that may improve the quality and maturity of the iHCs. In addition, transcriptional characterization of older hair cells will allow for identification of additional TFs, which may improve the reprogramming strategy. Second, our current strategy relies on constitutive expression of the reprogramming factors, while continuous expression of *Atoh1* is known to halt hair cell maturation (Liu et al., 2012b; Liu et al., 2014a). We plan to overcome this limitation by using inducible gene expression constructs to drive reprogramming in the future. Finally, lack of organ-specific context in vitro may not provide additional signals for maturation. In fact, we demonstrate that iHCs co-cultured with dissociated organ of Corti cells, promoted morphological and electrophysiological maturation of iHCs (Figure 6). This functional maturation may be mediated, at least in part, by improved trafficking and assembly of ion channel subunits conferred by the co-cultures. We plan to examine the transcriptional profile changes that occur in iHCs after co-culture in order to understand what genes may be contributing to the morphological and functional maturation.

As has been documented in other reprogramming experiments (Wapinski et al., 2013; Wapinski et al., 2017; Rhee et al., 2017), the chromatin landscape was also drastically remodeled during reprogramming of MEFs to a hair cell-like state, readily opening de novo distal elements to change their chromatin to resemble P1 cochlear hair cells. Similar to the RNA sequencing results, there is a residual MEF signature of the chromatin landscape, and the failure to close down these chromatin regions may be acting as a barrier for more efficient and/or faithful reprogramming. In addition, the opening of a large number of peaks inappropriately (peaks that occur in neither MEFs of primary P1 hair cells) suggests that our cocktail may lack a transcriptional repressor, and that these inappropriately open distal elements may provide some explanation for the low level of inappropriate gene expression (Figures 2 and 4).

Despite these pitfalls, common to most if not all reprogramming strategies published to date (Ieda et al., 2010; Son et al., 2011; Treutlein et al., 2016; Kaminski et al., 2016; Van Pham et al., 2017), induced hair cells are highly similar to their primary counterparts on transcriptional and epigenetic levels, as well as functional levels.

### Direct reprogramming as a strategy for gene therapy and identification of genetic causes of hearing loss

Recently the use of Anc80-based adeno associated vectors made inner ear gene delivery feasible (Suzuki et al., 2017; Tao et al., 2018; Tan et al., 2019). The proof of concept studies have demonstrated functional recovery after administration of TMC1 gene therapy in animals carrying a mutation in the gene (Yoshimura et al., 2019). Yet, the genetic causes of deafness are often unknown in patients, and loss of hair cells remains the leading contributor to hearing loss worldwide. We found that the SAPG four transcription factor combination is significantly more effective at activating the expression of hair cell genes MyosinVIIa and Parvalbumin in adult tail tip fibroblasts, and postnatal (P8) supporting cells, when compared to *Atoh1* alone (Figure 5). Supporting cells have been shown to be maintained in long deafened mice (Oesterle and Campbell, 2009) and humans (Johnsson et al., 1981). The demonstration that SAPG is able to convert P8 supporting cells to a hair cell-like fate, highlights the potential for future gene therapy approaches for hearing loss.

The ability to reprogram cells to a hair cell fate provides new opportunities to target hearing loss through the development of disease-specific drug screens and personalized medicine. Primary human fibroblasts taken from patients, can be reprogrammed to induced pluripotent stem cells that can then be expanded and reprogrammed to a hair cell fate for patient- and disease-specific studies, opening the opportunity to study hearing loss mutations, ototoxicity and regenerative medicine approaches (Koch et al., 2011; Lim et al., 2016a; Lim et al., 2016b; Shi et al., 2018; Huang et al., 2019; Villanueva-Paz et al., 2019; Lee et al., 2019).

### Reprogramming strategy vs other approaches

The evaluation of ototoxic compounds, and the identification of new otoprotectants has been severely limited by the lack of sufficient numbers of mammalian hair cells available for study. Although directed differentiation of ESCs towards the sensory hair cells have been described (Li et al., 2003; Oshima et al., 2010; Koehler et al., 2013; Ronaghi et al., 2014), these three-dimensional protocols are time consuming and the outcomes are ESC-line dependent with variable efficiency across protocols (Hiler et al., 2015; Mellough et al., 2019; Yoon et al., 2019). Direct reprogramming allows for a much faster and more reliable approach to produce large quantities of induced hair cell-like cells to scale, and the monolayer culture used here overcomes several of the short-comings of directed differentiation systems presented so far, such as long culture periods (Koehler et al., 2013), and 3D cultures that are more difficult to image robotically (Breslin and O'Driscoll, 2013; van Vliet et al., 2014). The efficiency and reproducibility of direct reprogramming is essential for high throughput screening approaches. We demonstrated that iHCs are selectively vulnerable to gentamicin, and can be reprogrammed and cultured in microtiter plate format, providing a robotic-imaging platform for scalable monitoring of iHC survival and small-molecule screening. Importantly, reprogramming does not require an iPSC intermediate, thus generating iHCs from human patients with known or novel genetic mutations associated with hearing loss will enable screening for new therapeutic targets and agents for the treatment of genetic causes of deafness.

The direct lineage conversion of somatic cells to a hair cell-like fate provides the means to study many outstanding questions in inner ear biology. For instance, the nature of gene regulatory logic underlying the differentiation of the variety of cochlear and vestibular hair cell types, as well as mechanisms underlying hair cell degeneration caused by ototoxins, and the numerous mutations responsible for the many types of syndromic and non-syndromic hearing loss.

## Materials and methods

### Contacts for reagent and resource sharing

Further information and requests for resources and reagents used in this study should be directed to the Lead Contacts, Dr. Justin Ichida (ichida@usc.edu) or Dr. Neil Segil (nsegil@med.usc.edu).

### Mice

All experiments were performed at the University of Southern California. All animal experiments were conducted according to the National Institutes of Health Guide for Care and Use of Laboratory Animals. Protocols and experiments using animals were approved by the Institutional Animal Care and Use Committee at the University of Southern California.

Mice were housed with free access to chow and water and a 12 hr day/night cycle. Breeding and genotyping of the mice was performed according to USC standard procedures.

*Atoh1*-nGFP (previously known as Math1-GFP) transgenic line was obtained from Jane Johnson. *Atoh1*-nGFP transgenic mice were mated with wild type CD1 mice to obtain litters for mouse embryonic fibroblast isolations and tail tip fibroblast isolations. Wild type CD1 mice were used for harvesting wild type organs of Corti in co-culture experiments. Lfng-CreERt2::Rosa26^tdTomato^ transgenic mice were used for harvesting organs of Corti with lineage traced supporting cells.

### Molecular cloning of viral plasmids and virus production

Complimentary DNAs for the 16 candidate transcription factors were each cloned into viral expression vectors using the Gateway cloning (Invitrogen). Retroviral and lentiviral plasmids were constructed into the entry vector pDONR221. Entry clones were recombined into destination vectors via LR reaction into the pMXS-DEST (retro) or FUWO-tetO-DEST (lenti).

Plat-E cells and HEK293 cells were cultured in MEF medium (DMEM containing 10% fetal bovine serum) and used to produce retroviruses and lentiviruses respectively. Cells were transfected at 90% confluency with viral vectors containing genes of interest and viral packaging plasmids (PIK-MLV-gp and pHDM for retrovirus; pPAX2 and VSVG for lentivirus) using linear polyethylenimine (PEI) (Sigma-Aldrich). After 24 hr of incubation with the plasmid DNA and PEI, the medium was replaced with fresh MEF medium and the culture was continued. Supernatants from the transfected cells were collected at 24 hr and 48 hr after medium replacement, filtered through 0.45 um filters and used immediately if generated from Plat-E or concentrated using Lenti-X concentrator (Clontech) and stored at −80°C if generated from HEK293T.

MEFs were transduced by mixing virus with MEF media. The virus containing media was removed from the MEFs after 24 hr and replaced with MEF media. The next day, medium was switched to hair cell medium (HCM: DMEM/F-12 (supplier), N2 and B27 supplements (supplier), EGF (2.5 ng/ml), and FGF (5 ng/ml)).

### MEF isolation

Mouse embryonic fibroblasts (MEFs) were obtained from E13-14 embryos taking care to exclude contamination with other Atoh1 expressing tissues (kidney, brain, spinal cord and webbing between digits). Tissue was minced with a razor blade and enzymatically dissociated with 0.25% trypsin-EDTA for 30 min at 37°C. Trypsinization was quenched by addition of MEF media (previously described). The isolated cells were centrifuged (800 g for 10 min) and the pellet was resuspended in MEF media before plating onto gelatin coated T75 tissue culture flasks. We found that plating two embryos per T75 gave optimal survival post-dissection. The MEFs were cultured until confluency was reached and then cryopreserved in liquid nitrogen using freezing media (1:1 mixture of MEF media and Freezing media (FM; 80% fetal bovine serum and 20% DMSO)). MEFs were used without further passaging for reprogramming experiments. All cells were tested for mycoplasma contamination and came back negative.

### TTF isolation

Tail tip fibroblasts (TTFs) were obtained from 6 month old adult Atoh1-nGFP transgenic mice. The tail was harvested from the sacrificed mouse by removing the skin and dissecting the remaining tail tissue into small segments. After plating on gelatin coated dishes, the tissue adhered to the dish and the expanding cells eventually covered the dish. The TTFs were harvested for reprogramming by simply moving the segments to a new dish and then collecting the remaining adherent cells. TTFs were cultured in DMEM with 40% fetal bovine serum. TTFs were frozen in liquid nitrogen and used without further passaging for reprogramming experiments. All cells were tested for mycoplasma contamination and came back negative.

### Merkel cell isolation

Skin was obtained from postnatal day 1 (P1) mice. Skin was incubated overnight at 4°C in Accutase. The epithelium (epidermis and hair follicles) was separated from the underlying dermis with forceps and the epidermal cells were dissociated with trypsin for 10 min at 37°C then dissociated to single cell suspension. The freshly isolated epidermal cell suspension was then FACS purified to sort for *Atoh1*-nGFP positive Merkel cells.

### Gut secretory cell isolation

Small intestines were obtained from adult mice. Intestinal villi were scraped away, crypt epithelium was collected by shaking in 5 mM EDTA for 50 min at four degrees Celsius, and single cell suspensions were prepared by digestion in 4x TrypLE (Invitrogen) for 50 min at 37°C. The freshly isolated cell suspension was then FACS purified to sort for *Atoh1*-nGFP positive cells gut secretory cells.

### Cerebellar granule precursor isolation

Cerebellums were obtained from postnatal day 1 (P1) mice. Tissue was minced and enzymatically digested using 0.25% trypsin for 10 min at 37°C then dissociated to single cell suspension. The freshly isolated cerebellar cell suspension was then FACS purified to sort for *Atoh1*-nGFP positive cells cerebellar granule precursors.

### Primary hair cell culture

The primary hair cell culture was established by dissecting the organs of Corti from P1 transgenic *Atoh1*-nGFP mice. The cells were dissociated to a single cell suspension and plated onto laminin coated tissue culture plates or cover slips.

The primary cultures from Lfng-CreERt2::Rosa26^tdTomato^ transgenic mice were done at postnatal day 8 (P8). Lfng-CreERt2::Rosa26^tdTomato^ transgenic mice were injected with tamoxifen at postnatal day three for lineage tracing of the Lfng+ supporting cell population. The organs of Corti were harvested at P8, dissociated to single cell suspension in HCM and the reprogramming factors were added to the cell suspension. The cells were then plated onto laminin coated tissue culture treated cover slips. The virus containing media was replaced after 24 hr with fresh HCM.

All primary cultures were plated using ROCK Inhibitor (Y-27632) (Sigma-Aldrich) for the first 24 hr to help promote survival.

### Co-culture of iHCs

Induced hair cells were FACS purified to obtain the *Atoh1*-nGFP positive cells and collected in HCM. The primary organ of Corti was dissected from wild type mice at E13.5 and enzymatically dissociated to a single cell suspension containing primary hair cells, primary supporting cells and a portion of the surrounding periotic mesenchmye. The iHCs were then mixed with the dissociated organs of Corti. The ratio of iHC to cells of the organ of Corti was kept at about 1:33. This ratio was determined from the fact that the primary organ of Corti contains approximately 3000 hair cells and upon dissociation gives approximately 100,000 total cells. Co-cultures were grown on tissue culture treated coverslips in wells of 24 well plates that had been coated with a 20 ul drop of matrigel (10% in HCM) at the center of the coverslip. The co-culture cell suspension was plated as 30 ul drops (2,500–3,000 cells per ul) in the center of the matrigel coated drop on the cover slip. 12–24 hr after plating the drops the well was flooded with 500 ul of HCM. All cocultures were maintained in HCM.

### Immunostaining

Cells for staining were washed with PBS and fixed using 4% paraformaldehyde (PFA) in phosphate-buffered saline (PBS) for 15 min at room temperature. For permeabilization and blocking the cells were incubated in PBST (0.1% Triton-X 100 in PBS) with 10% fetal bovine serum for 2 hr at room temperature or overnight at 4°C. After blocking, the cells were washed 3 times for 5 min with PBS. Cells were then incubated with the primary antibody overnight at 4°C. Then the cells were washed with PBS again before incubation with the secondary antibody for one hour at room temperature or overnight at 4°C. Primary and secondary antibodies were diluted in PBST with 10% serum. The DNA was stained with Hoechst diluted 1:1000 in PBS for 10 min at room temperature.

### Antibodies

Anti-Parvalbumin (Sigma-Aldrich, catalog# P3088-100UL)
Anti-Espin (Gift from Hudspeth Lab)
Anti-MyosinVIIa (Proteus Bioscience, catalog# 25–6790)
Molecular Probes Phalloidin Rhodamine (Thermo Fisher Scientific, catalog# R415)
Phalloidin-iFluor 647 Reagent (Abcam Biochemicals, catalog# ab176759)
Anti-acetylated Tubulin (Sigma Aldrich, catalog# T7451-100UL)
Anti-Sox2 (Abcam, catalog# ab97959)

### Imaging

Immunostaining images of adherent cell cultures were acquired on an LSM780 confocal microscope using Carl Zeiss Zen blue/black software and processed using Adobe Illustrator CS6 software. For quantification of reprogramming efficiency in adherent cultures, images were acquired at 10x using the Molecular Devices ImageExpress. The images were either processed manually using ImageJ software and the Cell Counter plug in or automatically using SVCell RS (described below). Counts are represented as reprogramming efficiency (percent of *Atoh1*-nGFP+ cells per well of 5000 MEFs infected).

### iHC detection and counting method

Automated cell counting used thresholds for size, intensity and roundness of the *Atoh1-*nGFP signal. The imaging was done at 10x. For each time frame, the customized time-lapse nuclei count recipe of SVCell RS is applied to first reduce noise with image smoothing. Objects are detected by performing background removal followed by adaptive thresholding. A size filtering is then applied to remove objects that are either too large or too small. The count of remaining objects is measured for the time point. Batch processing is available for applying the recipe to multiple time-lapse images and saving results. To ensure the reliability of the automated counting a comparison was done of 20 wells counted both manually and automatically (p=0.53).

### Flow cytometry preparations

Primary hair cells were harvested from *Atoh1*-nGFP transgenic mice. The cochleas were incubated in 0.25% trypsin for 8 min and gently triturated to single cell suspension. Media (DMEM with 10% FBS) was added to the dissociated cells and then spun down at 1000 rpm for 5 min, resuspended in Hair Cell Media, passed through a 70 um cell strainer and then FACS-purified). The same procedure was used to FACS-purify dsRED MEFs and *Atoh1*-nGFP+ cells from the reprogramming cultures.

### RNA sequencing

Total RNA was extracted from primary mouse hair cells (at postnatal day 1), mouse iHCs (at day 14 post infection with reprogramming factors) and MEFs (at 14 days post transduction with dsRed). For each replicate 20,000 FACS-sorted cells were used as input for RNA-seq. Total RNA was extracted with either Quick-RNA Microprep kit (Zymo Research), quantified by bioanalyzer and then processed for libraries with either QIAseq FX Single Cell RNA Library Kit (Qiagen) or TruSeq RNA Library Prep Kit v2 (Illumina). Specific sequencing parameters and instrument models were submitted with GEO datasets. At least three replicates were collected for each condition and sequenced to a depth of at least 20 million reads.

Reads were mapped to the mouse reference genome (Gencode Mm10v11) using STAR. Read counts were quantified by RSEM. Only protein coding polyA tail transcripts and autosomal genes were kept. Transcript counts were collapsed to gene counts. Differentially expressed genes were identified using the DESeq2 package. Genes with a log fold change threshold greater than one and adjusted P-value of less than 0.1 were considered significant. Principle component analysis and unsupervised hierarchical clustering of RNA-seq was performed using counts transformed by DESeq2’s regularized logarithm (Rlog).

GEO accession number: GSE149260.

### GO analysis

Gene ontology analysis was performed on categorized gene sets using R clusterProfiler package. GO results were visualized using the R enrichplot package.

### GSEA analysis

Gene Set Enrichment Analysis was performed using the R package fgsea. The Wald statistic from the differential comparison of reprogrammed cells versus MEFs was used to pre-rank genes for subsequent GSEA analysis. Gene sets representing unique signatures for each Atoh1 positive cell-type were tested for enrichment in SAPG. To determine signature gene sets for each Atoh1 cell type, only genes with a log2 foldchange greater than or equal to two with adjusted P-value less than 0.01 compared between each profiled Atoh1 positive cell-type were used. Utricle and cochlear hair cells were treated as a single cell type due to small number of unique genes at the postnatal day one developmental stage used.

### ATAC-seq

Cells were collected by FACS purification into cold PBS, and centrifuged 500 xg for 15 min. Cell pellet were resuspended with 50 ul transposition buffer consisting of 10 mM Tris-HCL pH8.0, 5 mM MgCl2, 10% DMF, 0.2% NP40, and home-made transposase Tn5. Transposition was performed at 37°C for 20 min. DNA was collected immediately after transposition using Qiagen Mini-elute kit.

Encode pipeline was adapted for alignment and QC for ATAC-seq and ChIP-seq data. Paired-end reads were quality trimmed with cutadapt (v1.18) and aligned to mouse reference genome (Gencode Mm10v11) with bowtie2 (v2.2.6) using parameters -X2000 -mm –local. PCR duplicates were removed based on genomic coordinates. Only autosomal chromosomes were selected and used for downstream analysis.

Specific sequencing parameters and instrument models were submitted with GEO datasets.

### ChIP-seq

Histone ChIP-seq protocol was developed by us based on μChIP-PCR protocol published previously (Stojanova et al., 2016) with additional Tn5 tagmentation step. Briefly, chromatin was cross-linked with 1% formaldehyde (Thermo Fisher) for 8 min, quenched with 125 mM Glycine (Sigma) for 5 min at room temperature, sonicated using the microtip of a High Intensity Ultrasonic Processor (Sonics and Materials, Newtown, CT; amplitude 50, power 50) for 8 × 30 s with 30 s pause, tagmentated with Tn5 transposase for 30 min at 37°C, incubated with antibody complexed with Dynabeads Protein A (Thermo Fisher) overnight at 4°C, precipitated and washed three times on magnetic rack, and finally PCR amplified with primers matching Tn5 adapters.

Encode pipeline was adapted for alignment and QC for ChIP-seq data. Paired-end reads were quality trimmed to 36 bp with cutadapt (v1.18) and aligned to mm10 reference genome (Gencode Mm10v11) with STAR aligner using parameters end-to-end and alignIntronMax = 1 for DNA alignment. PCR duplicates were removed with STAR. Only autosomal chromosomes were selected and used for downstream analysis.

Specific sequencing parameters and instrument models were submitted with GEO datasets.

### Chromatin analysis

ATAC peaks and H3K27ac peaks were identified using the R package chromstaR (parameters: binsize = 500 bp, stepsize = 250 bp, mode = full). An equal number of reads were randomly sampled for H3K27ac replicates (17.5 million) and ATAC replicates (15 million reads) as input for subsequent chromatin analysis. For peak calling, a false discovery rate (FDR) cutoff of 0.01 and 0.001 was used for ATAC and H3K27ac respectively and an RPKM cut off >2. Promoter regions were defined by 2 kb upstream of 500 bp downstream of protein coding transcription start sites; all remaining regions were considered distal. Enhancers were defined by cooccurrence of an ATAC peak and H3K27ac peak at distal regions.

Differential ATAC peak analysis was performed between P1HC, SAPG iHCs, and MEFs using chromstaR. Regions with a differential score of at least 0.999999 were considered differentially accessible. Regions with differential score less than 1E-06 were considered non-differentially accessible.

Deeptools was used to average replicates and calculate coverage tracks and for ATAC-seq and ChIP-seq data for visualization on IGV and heatmaps.

### Electrophysiology

Whole cell patch clamping was performed on three different preparations of cells. The first preparation was iHCs in the monolayer culture of MEFs at D14-15 post infection with SAPG. The second preparation was iHCs FACS purified and replated with dissociated wild type organ of Corti. The third preparation was postnatal day one primary hair cells from the dissociated transgenic *Atoh1*-nGFP organ of Corti. Preparations were viewed at X630 using a Zeiss Axios Examiner D1 microscope fitted with Zeiss W Plan-Aprochromat optics. Signals were driven, recorded, and amplified with an Multiclamp 700B amplifier, Digidata 1440 board and pClamp 10.7 software (pClamp, RRID:SCR_011323). Recording and cleaning pipettes were fabricated using filamented borosilicate glass. Pipettes were fired polished to yield an access resistance between 4–8 MΩ. Each recording pipette was covered in a layer of parafilm to reduce pipette capacitance. Recording pipettes were filled with standard internal solution. The contents of the standard internal solution are (in mM): 135 KCl, 3.5 MgCl2, 3 Na2ATP, 5 HEPES, 5 EGTA, 0.1 CaCl2, 0.1 Li-GTP, and titrated with 1M KOH to a pH of 7.35 and an osmolarity of about 300 mmol/kg. The voltage clamp protocol was performed by holding the cell at −60 mV followed by a stimulus of voltage steps (−120 to +70 mV, by intervals of 10 mV). The current response of the cell was recorded along with measures of ionic current peak amplitudes, channel conductance values, and current activation kinetics.

Analysis of the data was performed using a combination of pClamp (pClamp, RRID:SCR_011323), Matlab (MATLAB, RRID:SCR_001622), JMP (JMP, RRID:SCR_014242), Origin Pro (OriginPro, RRID:SCR_015636), and Imaris (Imaris, RRID:SCR_007370). pClamp software was be used to gather and quantify raw data from electrophysiological recordings.

### FM lipophilic styryl dye uptake assay

Cells were incubated with 1 uM FM 4-64FX, the fixable analog of FM4-64 (Life Technologies, catalog# F34653). Prior to incubation the FM 4-64FX was resuspended in ice cold HBSS at a 1 mM concentration. The cells were incubated with a final concentration of 1 uM FM 4-64FX in ice cold HBSS for 30 s. After incubation, the cells were rinsed in HBSS three times and then immediately imaged. Using the software ImageJ, the images were filtered on minimum background intensity in order to reduce the amount of background signal. The filter measures the minimum signal intensity found in the image and applies the filter to remove the minimum signal across the entire image. This image enhancement was used uniformly on all images and all channels for each cell type.

### GTTR uptake assay

Gentamicin sulfate salt (Sigma Aldrich catalog# G3632-5G, 50 mg/ml in K2CO3, pH0) and Texas-Red (Thermo Fisher Scientific catalog# T20175, 2 mg/ml in dimethyl formamide) were agitated together overnight to produce gentamicin-Texas Red conjugate (GTTR). The mixture contained 4.4mls of 50 mg/ml gentamicin (GT) with 0.6mls of 2 mg/ml Texas Red (TR) to produce approximately 300:1 molar ratio of GT:GTTR. A high ratio of gentamicin ensures a minimum of unbound Texas Red molecules. The molecular weight of GT is 477.6 g/mol and the molecular weight of TR is 816.94 g/mol. The GTTR was made at a stock concentration of 100 mM. The cells were incubated with HCM containing 0.5 mM or 1 mM GTTR for three hours. After incubation the cells were washed three times with PBS and then immediately fixed using 4% PFA in PBS for 15 min at room temperature.

### Ototoxicity assay

The cells were cultured (for primary hair cells) or reprogrammed (for iHCs and iMNs) in a 96 well tissue culture plate. The primary hair cells were used 24 hr post dissociation of the organ of Corti and plating. The iHCs and iMNs were reprogrammed for 14 days prior to starting the survival assay. The gentamicin was dissolved in HCM at a concentration of 100 mM and subsequently diluted to 8 mM in HCM. The stock at 8 mM in HCM was used for serial dilution to get the desired range of concentrations (8 mM, 4 mM, 2 mM, 1 mM, 0.5 mM, 0.25 mM, and 0.125 mM). The control wells received only HCM.

The assay was performed over a period of 5 days. The HCM containing with or without gentamicin was added to the cells on Day one and the cells were imaged every 24 hr after the initial treatment. Subsequent media changes were performed every other day (Day 3). The HCM for gentamicin treated wells and control wells was made fresh for each media change. The assay ended on Day 5. The Molecular Devices ImageExpress was used for imaging the plate robotically every 24 hr. The images were taken at 10x.

### Statistics

Sample numbers, experimental repeats and statistical test used are indicated in figure legends. Unless otherwise stated, data presented as mean + SEM of at least three biological replicates. Significance summary: p>0.05 (ns), ∗p≤0.05, ∗∗p≤0.01, ∗∗∗p≤0.001, and ∗∗∗∗p≤0.0001.

## Data Availability

Sequencing data have been deposited in GEO (accession number GSE149260). Sequence data associated with this paper can be visualized on the gEAR website (https://umgear.org/p?l=e2d98834). The following dataset was generated: MenendezLTrecekTGopalakrishnanSTaoTMarkowitzALYuHZWangXELlamasJHuangCLeeJKalluriRIchidaJSegilN2020Generation of Inner Ear Hair Cells by Direct Lineage Conversion of Primary Somatic CellsNCBI Gene Expression OmnibusGSE14926010.7554/eLife.55249PMC732649332602462

## Additional files

Transparent reporting form

## References

[bib1] Abdolazimi Y, Stojanova Z, Segil N (2016). Selection of cell fate in the organ of Corti involves the integration of hes/Hey signaling at the *Atoh1* promoter. Development.

[bib2] Ahmed M, Wong EY, Sun J, Xu J, Wang F, Xu PX (2012). Eya1-Six1 interaction is sufficient to induce hair cell fate in the cochlea by activating Atoh1 expression in cooperation with Sox2. Developmental Cell.

[bib3] Al-Malky G, Dawson SJ, Sirimanna T, Bagkeris E, Suri R (2015). High-frequency audiometry reveals high prevalence of aminoglycoside ototoxicity in children with cystic fibrosis. Journal of Cystic Fibrosis.

[bib4] Alharazneh A, Luk L, Huth M, Monfared A, Steyger PS, Cheng AG, Ricci AJ (2011). Functional hair cell mechanotransducer channels are required for aminoglycoside ototoxicity. PLOS ONE.

[bib5] Atkinson PJ, Huarcaya Najarro E, Sayyid ZN, Cheng AG (2015). Sensory hair cell development and regeneration: similarities and differences. Development.

[bib6] Babos KN, Galloway KE, Kisler K, Zitting M, Li Y, Shi Y, Quintino B, Chow RH, Zlokovic BV, Ichida JK (2019). Mitigating antagonism between transcription and proliferation allows Near-Deterministic cellular reprogramming. Cell Stem Cell.

[bib7] Bardhan T, Jeng JY, Waldmann M, Ceriani F, Johnson SL, Olt J, Rüttiger L, Marcotti W, Holley MC (2019). Gata3 is required for the functional maturation of inner hair cells and their innervation in the mouse cochlea. The Journal of Physiology.

[bib8] Bermingham NA, Hassan BA, Price SD, Vollrath MA, Ben-Arie N, Eatock RA, Bellen HJ, Lysakowski A, Zoghbi HY (1999). Math1: an essential gene for the generation of inner ear hair cells. Science.

[bib9] Bodmer D (2008). Protection, regeneration and replacement of hair cells in the cochlea: implications for the future treatment of sensorineural hearing loss. Swiss Medical Weekly.

[bib10] Boëda B (2002). Myosin VIIa, harmonin and cadherin 23, three usher I gene products that cooperate to shape the sensory hair cell bundle. The EMBO Journal.

[bib11] Bohne BA, Harding GW (2000). Degeneration in the cochlea after noise damage: primary versus secondary events. The American Journal of Otology.

[bib12] Bramhall NF, Shi F, Arnold K, Hochedlinger K, Edge AS (2014). Lgr5-positive supporting cells generate new hair cells in the postnatal cochlea. Stem Cell Reports.

[bib13] Breslin S, O'Driscoll L (2013). Three-dimensional cell culture: the missing link in drug discovery. Drug Discovery Today.

[bib14] Brignull HR, Raible DW, Stone JS (2009). Feathers and fins: non-mammalian models for hair cell regeneration. Brain Research.

[bib15] Buenrostro JD, Wu B, Chang HY, Greenleaf WJ (2015a). Atac‐seq: a method for assaying chromatin accessibility genome‐wide. Current Protocols in Molecular Biology.

[bib16] Buenrostro JD, Wu B, Litzenburger UM, Ruff D, Gonzales ML, Snyder MP, Chang HY, Greenleaf WJ (2015b). Single-cell chromatin accessibility reveals principles of regulatory variation. Nature.

[bib17] Burns JC, Kelly MC, Hoa M, Morell RJ, Kelley MW (2015). Single-cell RNA-Seq resolves cellular complexity in sensory organs from the neonatal inner ear. Nature Communications.

[bib18] Cai T, Seymour ML, Zhang H, Pereira FA, Groves AK (2013). Conditional deletion of Atoh1 reveals distinct critical periods for survival and function of hair cells in the organ of corti. Journal of Neuroscience.

[bib19] Cai T, Jen H-I, Kang H, Klisch TJ, Zoghbi HY, Groves AK (2015). Characterization of the transcriptome of nascent hair cells and identification of direct targets of the Atoh1 transcription factor. Journal of Neuroscience.

[bib20] Chardin S, Romand R (1995). Regeneration and mammalian auditory hair cells. Science.

[bib21] Chen P, Johnson JE, Zoghbi HY, Segil N (2002). The role of Math1 in inner ear development: uncoupling the establishment of the sensory primordium from hair cell fate determination. Development.

[bib22] Chen X, Shen Y, Draper W, Buenrostro JD, Litzenburger U, Cho SW, Satpathy AT, Carter AC, Ghosh RP, East-Seletsky A, Doudna JA, Greenleaf WJ, Liphardt JT, Chang HY (2016). ATAC-see reveals the accessible genome by transposase-mediated imaging and sequencing. Nature Methods.

[bib23] Chen P, Segil N (1999). p27(Kip1) links cell proliferation to morphogenesis in the developing organ of corti. Development.

[bib24] Cheng AG, Cunningham LL, Rubel EW (2005). Mechanisms of hair cell death and protection. Current Opinion in Otolaryngology & Head and Neck Surgery.

[bib25] Chonko KT, Jahan I, Stone J, Wright MC, Fujiyama T, Hoshino M, Fritzsch B, Maricich SM (2013). Atoh1 directs hair cell differentiation and survival in the late embryonic mouse inner ear. Developmental Biology.

[bib26] Corwin JT, Cotanche DA (1988). Regeneration of sensory hair cells after acoustic trauma. Science.

[bib27] Costa A, Sanchez-Guardado L, Juniat S, Gale JE, Daudet N, Henrique D (2015). Generation of sensory hair cells by genetic programming with a combination of transcription factors. Development.

[bib28] Costa A, Powell LM, Lowell S, Jarman AP (2017). Atoh1 in sensory hair cell development: constraints and cofactors. Seminars in Cell & Developmental Biology.

[bib29] Cotanche DA, Corwin JT (1991). Stereociliary bundles reorient during hair cell development and regeneration in the chick cochlea. Hearing Research.

[bib30] Cox BC, Chai R, Lenoir A, Liu Z, Zhang L, Nguyen DH, Chalasani K, Steigelman KA, Fang J, Rubel EW, Cheng AG, Zuo J (2014). Spontaneous hair cell regeneration in the neonatal mouse cochlea in vivo. Development.

[bib31] Creyghton MP, Cheng AW, Welstead GG, Kooistra T, Carey BW, Steine EJ, Hanna J, Lodato MA, Frampton GM, Sharp PA, Boyer LA, Young RA, Jaenisch R (2010). Histone H3K27ac separates active from poised enhancers and predicts developmental state. PNAS.

[bib32] Dallos P (1985). Membrane potential and response changes in mammalian cochlear hair cells during intracellular recording. The Journal of Neuroscience.

[bib33] Demêmes D, Eybalin M, Renard N (1993). Cellular distribution of parvalbumin immunoreactivity in the peripheral vestibular system of three rodents. Cell and Tissue Research.

[bib34] Doetzlhofer A, White PM, Johnson JE, Segil N, Groves AK (2004). In vitro growth and differentiation of mammalian sensory hair cell progenitors: a requirement for EGF and periotic mesenchyme. Developmental Biology.

[bib35] Doetzlhofer A, Basch ML, Ohyama T, Gessler M, Groves AK, Segil N (2009). Hey2 regulation by FGF provides a Notch-independent mechanism for maintaining pillar cell fate in the organ of corti. Developmental Cell.

[bib36] Driver EC, Sillers L, Coate TM, Rose MF, Kelley MW (2013). The Atoh1-lineage gives rise to hair cells and supporting cells within the mammalian cochlea. Developmental Biology.

[bib37] Eybalin M, Ripoll C (1990). Immunolocalization of parvalbumin in two glutamatergic cell types of the guinea pig cochlea: inner hair cells and spinal ganglion neurons]. Comptes Rendus De l'Academie Des Sciences. Serie III, Sciences De La Vie.

[bib38] Fekete DM, Muthukumar S, Karagogeos D (1998). Hair cells and supporting cells share a common progenitor in the avian inner ear. The Journal of Neuroscience.

[bib39] Forge A, Li L, Nevill G (1998). Hair cell recovery in the vestibular sensory epithelia of mature guinea pigs. The Journal of Comparative Neurology.

[bib40] Gale JE, Marcotti W, Kennedy HJ, Kros CJ, Richardson GP (2001). FM1-43 dye behaves as a permeant blocker of the hair-cell mechanotransducer channel. The Journal of Neuroscience.

[bib41] Géléoc GS, Holt JR (2003). Developmental acquisition of sensory transduction in hair cells of the mouse inner ear. Nature Neuroscience.

[bib42] Géléoc GS, Holt JR (2014). Sound strategies for hearing restoration. Science.

[bib43] Goetze S, Mateos-Langerak J, Gierman HJ, de Leeuw W, Giromus O, Indemans MH, Koster J, Ondrej V, Versteeg R, van Driel R (2007). The three-dimensional structure of human interphase chromosomes is related to the transcriptome map. Molecular and Cellular Biology.

[bib44] Gopalakrishnan S, Hor P, Ichida JK (2017). New approaches for direct conversion of patient fibroblasts into neural cells. Brain Research.

[bib45] Groves AK (2010). The challenge of hair cell regeneration. Experimental Biology and Medicine.

[bib46] Hasson T, Gillespie PG, Garcia JA, MacDonald RB, Zhao Y, Yee AG, Mooseker MS, Corey DP (1997). Unconventional myosins in inner-ear sensory epithelia. Journal of Cell Biology.

[bib47] Heintzman ND, Stuart RK, Hon G, Fu Y, Ching CW, Hawkins RD, Barrera LO, Van Calcar S, Qu C, Ching KA, Wang W, Weng Z, Green RD, Crawford GE, Ren B (2007). Distinct and predictive chromatin signatures of transcriptional promoters and enhancers in the human genome. Nature Genetics.

[bib48] Hiler D, Chen X, Hazen J, Kupriyanov S, Carroll PA, Qu C, Xu B, Johnson D, Griffiths L, Frase S, Rodriguez AR, Martin G, Zhang J, Jeon J, Fan Y, Finkelstein D, Eisenman RN, Baldwin K, Dyer MA (2015). Quantification of retinogenesis in 3D cultures reveals epigenetic memory and higher efficiency in iPSCs derived from rod photoreceptors. Cell Stem Cell.

[bib49] Hinojosa R, Nelson EG, Lerner SA, Redleaf MI, Schramm DR (2001). Aminoglycoside ototoxicity: a human temporal bone study. The Laryngoscope.

[bib50] Hoa M, Olszewski R, Li X, Taukulis I, Gu S, DeTorres A, Lopez IA, Linthicum FH, Ishiyama A, Martin D, Morell RJ, Kelley MW (2020). Characterizing adult cochlear supporting cell transcriptional diversity using Single-Cell RNA-Seq: validation in the adult mouse and translational implications for the adult human cochlea. Frontiers in Molecular Neuroscience.

[bib51] Holt JR, Pan B, Koussa MA, Asai Y (2014). TMC function in hair cell transduction. Hearing Research.

[bib52] Housley GD, Ashmore JF (1992). Ionic currents of outer hair cells isolated from the guinea-pig cochlea. The Journal of Physiology.

[bib53] Huang P, Sun L, Zhang L, Hui L (2019). Conversion of fibroblasts to hepatocytes in vitro. Methods in Molecular Biology.

[bib54] Hume CR, Bratt DL, Oesterle EC (2007). Expression of LHX3 and SOX2 during mouse inner ear development. Gene Expression Patterns.

[bib55] Ichida JK, Blanchard J, Lam K, Son EY, Chung JE, Egli D, Loh KM, Carter AC, Di Giorgio FP, Koszka K, Huangfu D, Akutsu H, Liu DR, Rubin LL, Eggan K (2009). A small-molecule inhibitor of tgf-Beta signaling replaces sox2 in reprogramming by inducing nanog. Cell Stem Cell.

[bib56] Ichida JK, Staats KA, Davis-Dusenbery BN, Clement K, Galloway KE, Babos KN, Shi Y, Son EY, Kiskinis E, Atwater N, Gu H, Gnirke A, Meissner A, Eggan K (2018). Comparative genomic analysis of embryonic, lineage-converted and stem cell-derived motor neurons. Development.

[bib57] Ieda M, Fu JD, Delgado-Olguin P, Vedantham V, Hayashi Y, Bruneau BG, Srivastava D (2010). Direct reprogramming of fibroblasts into functional cardiomyocytes by defined factors. Cell.

[bib58] Izumikawa M, Minoda R, Kawamoto K, Abrashkin KA, Swiderski DL, Dolan DF, Brough DE, Raphael Y (2005). Auditory hair cell replacement and hearing improvement by Atoh1 gene therapy in deaf mammals. Nature Medicine.

[bib59] Izumikawa M, Batts SA, Miyazawa T, Swiderski DL, Raphael Y (2008). Response of the flat cochlear epithelium to forced expression of Atoh1. Hearing Research.

[bib60] Jiang L, Jin R, Xu J, Ji Y, Zhang M, Zhang X, Zhang X, Han Z, Zeng S (2016). Hair cell regeneration or the expression of related factors that regulate the fate specification of supporting cells in the cochlear ducts of embryonic and posthatch chickens. Hearing Research.

[bib61] Johnsson LG, Hawkins JE, Kingsley TC, Black FO, Matz GJ (1981). Aminoglycoside-induced cochlear pathology in man. Acta Oto-Laryngologica.

[bib62] Kaminski MM, Tosic J, Kresbach C, Engel H, Klockenbusch J, Müller AL, Pichler R, Grahammer F, Kretz O, Huber TB, Walz G, Arnold SJ, Lienkamp SS (2016). Direct reprogramming of fibroblasts into renal tubular epithelial cells by defined transcription factors. Nature Cell Biology.

[bib63] Kawashima Y, Géléoc GS, Kurima K, Labay V, Lelli A, Asai Y, Makishima T, Wu DK, Della Santina CC, Holt JR, Griffith AJ (2011). Mechanotransduction in mouse inner ear hair cells requires transmembrane channel-like genes. Journal of Clinical Investigation.

[bib64] Kelley MW (2006). Regulation of cell fate in the sensory epithelia of the inner ear. Nature Reviews Neuroscience.

[bib65] Kelly MC, Chang Q, Pan A, Lin X, Chen P (2012). Atoh1 directs the formation of sensory mosaics and induces cell proliferation in the postnatal mammalian cochlea in vivo. Journal of Neuroscience.

[bib66] Kim TH, Li F, Ferreiro-Neira I, Ho LL, Luyten A, Nalapareddy K, Long H, Verzi M, Shivdasani RA (2014). Broadly permissive intestinal chromatin underlies lateral inhibition and cell plasticity. Nature.

[bib67] Klisch TJ, Xi Y, Flora A, Wang L, Li W, Zoghbi HY (2011). In vivo Atoh1 targetome reveals how a proneural transcription factor regulates cerebellar development. PNAS.

[bib68] Knight KR, Chen L, Freyer D, Aplenc R, Bancroft M, Bliss B, Dang H, Gillmeister B, Hendershot E, Kraemer DF, Lindenfeld L, Meza J, Neuwelt EA, Pollock BH, Sung L (2017). Group-Wide, prospective study of ototoxicity assessment in children receiving cisplatin chemotherapy (ACCL05C1): A report from the children's Oncology Group. Journal of Clinical Oncology.

[bib69] Koch P, Breuer P, Peitz M, Jungverdorben J, Kesavan J, Poppe D, Doerr J, Ladewig J, Mertens J, Tüting T, Hoffmann P, Klockgether T, Evert BO, Wüllner U, Brüstle O (2011). Excitation-induced ataxin-3 aggregation in neurons from patients with Machado-Joseph disease. Nature.

[bib70] Koehler KR, Mikosz AM, Molosh AI, Patel D, Hashino E (2013). Generation of inner ear sensory epithelia from pluripotent stem cells in 3D culture. Nature.

[bib71] Kozubek S, Lukásová E, Jirsová P, Koutná I, Kozubek M, Ganová A, Bártová E, Falk M, Paseková R (2002). 3d structure of the human genome: order in randomness. Chromosoma.

[bib72] Lalit PA, Salick MR, Nelson DO, Squirrell JM, Shafer CM, Patel NG, Saeed I, Schmuck EG, Markandeya YS, Wong R, Lea MR, Eliceiri KW, Hacker TA, Crone WC, Kyba M, Garry DJ, Stewart R, Thomson JA, Downs KM, Lyons GE, Kamp TJ (2016). Lineage reprogramming of fibroblasts into proliferative induced cardiac progenitor cells by defined factors. Cell Stem Cell.

[bib73] Langer T, am Zehnhoff-Dinnesen A, Radtke S, Meitert J, Zolk O (2013). Understanding platinum-induced ototoxicity. Trends in Pharmacological Sciences.

[bib74] Lee YS, Liu F, Segil N (2006). A morphogenetic wave of p27Kip1 transcription directs cell cycle exit during organ of corti development. Development.

[bib75] Lee M, Sim H, Ahn H, Ha J, Baek A, Jeon YJ, Son MY, Kim J (2019). Direct reprogramming to human induced neuronal progenitors from fibroblasts of familial and sporadic parkinson's Disease Patients. International Journal of Stem Cells.

[bib76] Leibovici M, Verpy E, Goodyear RJ, Zwaenepoel I, Blanchard S, Lainé S, Richardson GP, Petit C (2005). Initial characterization of Kinocilin, a protein of the hair cell kinocilium. Hearing Research.

[bib77] Lelli A, Asai Y, Forge A, Holt JR, Géléoc GS (2009). Tonotopic gradient in the developmental acquisition of sensory transduction in outer hair cells of the mouse cochlea. Journal of Neurophysiology.

[bib78] Lemon B, Tjian R (2000). Orchestrated response: a symphony of transcription factors for gene control. Genes & Development.

[bib79] Levine M, Tjian R (2003). Transcription regulation and animal diversity. Nature.

[bib80] Li H, Roblin G, Liu H, Heller S (2003). Generation of hair cells by stepwise differentiation of embryonic stem cells. PNAS.

[bib81] Li J, Zhang T, Ramakrishnan A, Fritzsch B, Xu J, Wong EYM, Loh Y-HE, Ding J, Shen L, Xu P-X (2020). Dynamic changes in cis-regulatory occupancy by Six1 and its cooperative interactions with distinct cofactors drive lineage-specific gene expression programs during progressive differentiation of the auditory sensory epithelium. Nucleic Acids Research.

[bib82] Liberman MC, Gao J, He DZZ, Wu X, Jia S, Zuo J (2002). Prestin is required for electromotility of the outer hair cell and for the cochlear amplifier. Nature.

[bib83] Lim SM, Choi B-O, Oh S-il, Choi WJ, Oh K-W, Nahm M, Xue Y, Choi JH, Choi JY, Kim Y-E, Chung KW, Fu X-D, Ki C-S, Kim SH (2016a). Patient fibroblasts-derived induced neurons demonstrate autonomous neuronal defects in adult-onset Krabbe disease. Oncotarget.

[bib84] Lim SM, Choi WJ, Oh KW, Xue Y, Choi JY, Kim SH, Nahm M, Kim YE, Lee J, Noh MY, Lee S, Hwang S, Ki CS, Fu XD, Kim SH (2016b). Directly converted patient-specific induced neurons mirror the neuropathology of FUS with disrupted nuclear localization in amyotrophic lateral sclerosis. Molecular Neurodegeneration.

[bib85] Liu J, Ashton MP, Sumer H, O'Bryan MK, Brodnicki TC, Verma PJ (2011). Generation of stable pluripotent stem cells from NOD mouse tail-tip fibroblasts. Diabetes.

[bib86] Liu Z, Owen T, Fang J, Srinivasan RS, Zuo J (2012a). In vivo notch reactivation in differentiating cochlear hair cells induces Sox2 and Prox1 expression but does not disrupt hair cell maturation. Developmental Dynamics.

[bib87] Liu Z, Dearman JA, Cox BC, Walters BJ, Zhang L, Ayrault O, Zindy F, Gan L, Roussel MF, Zuo J (2012b). Age-dependent in vivo conversion of mouse cochlear pillar and deiters' cells to immature hair cells by Atoh1 ectopic expression. Journal of Neuroscience.

[bib88] Liu Z, Fang J, Dearman J, Zhang L, Zuo J (2014a). In vivo generation of immature inner hair cells in neonatal mouse cochleae by ectopic Atoh1 expression. PLOS ONE.

[bib89] Liu H, Pecka JL, Zhang Q, Soukup GA, Beisel KW, He DZ (2014b). Characterization of transcriptomes of cochlear inner and outer hair cells. Journal of Neuroscience.

[bib90] Lo LC, Johnson JE, Wuenschell CW, Saito T, Anderson DJ (1991). Mammalian achaete-scute homolog 1 is transiently expressed by spatially restricted subsets of early neuroepithelial and neural crest cells. Genes & Development.

[bib91] Lowenheim H, Furness DN, Kil J, Zinn C, Gultig K, Fero ML, Frost D, Gummer AW, Roberts JM, Rubel EW, Hackney CM, Zenner H-P (1999). Gene disruption of p27Kip1 allows cell proliferation in the postnatal and adult organ of corti. PNAS.

[bib92] Lumpkin EA, Collisson T, Parab P, Omer-Abdalla A, Haeberle H, Chen P, Doetzlhofer A, White P, Groves A, Segil N, Johnson JE (2003). Math1-driven GFP expression in the developing nervous system of transgenic mice. Gene Expression Patterns.

[bib93] Maass JC, Gu R, Basch ML, Waldhaus J, Lopez EM, Xia A, Oghalai JS, Heller S, Groves AK (2015). Changes in the regulation of the notch signaling pathway are temporally correlated with regenerative failure in the mouse cochlea. Frontiers in Cellular Neuroscience.

[bib94] Marcotti W, Kros CJ (1999). Developmental expression of the potassium current _IK,n_ contributes to maturation of mouse outer hair cells. The Journal of Physiology.

[bib95] Marro S, Pang ZP, Yang N, Tsai MC, Qu K, Chang HY, Südhof TC, Wernig M (2011). Direct lineage conversion of terminally differentiated hepatocytes to functional neurons. Cell Stem Cell.

[bib96] Matei V, Pauley S, Kaing S, Rowitch D, Beisel KW, Morris K, Feng F, Jones K, Lee J, Fritzsch B (2005). Smaller inner ear sensory epithelia in neurog 1 null mice are related to earlier hair cell cycle exit. Developmental Dynamics.

[bib97] Matsui JI, Gale JE, Warchol ME (2004). Critical signaling events during the aminoglycoside-induced death of sensory hair cells in vitro. Journal of Neurobiology.

[bib98] McGrath J, Roy P, Perrin BJ (2017). Stereocilia morphogenesis and maintenance through regulation of actin stability. Seminars in Cell & Developmental Biology.

[bib99] Mellough CB, Collin J, Queen R, Hilgen G, Dorgau B, Zerti D, Felemban M, White K, Sernagor E, Lako M (2019). Systematic comparison of retinal organoid differentiation from human pluripotent stem cells reveals stage specific, cell line, and methodological differences. STEM CELLS Translational Medicine.

[bib100] Mistry BA, D'Orsogna MR, Chou T (2018). The effects of statistical multiplicity of infection on virus quantification and infectivity assays. Biophysical Journal.

[bib101] Mizutari K, Fujioka M, Hosoya M, Bramhall N, Okano HJ, Okano H, Edge AS (2013). Notch inhibition induces cochlear hair cell regeneration and recovery of hearing after acoustic trauma. Neuron.

[bib102] Oesterle EC, Campbell S (2009). Supporting cell characteristics in long-deafened aged mouse ears. Journal of the Association for Research in Otolaryngology.

[bib103] Oliver D, Knipper M, Derst C, Fakler B (2003). Resting potential and submembrane calcium concentration of inner hair cells in the isolated mouse cochlea are set by KCNQ-type potassium channels. The Journal of Neuroscience.

[bib104] Oshima K, Shin K, Diensthuber M, Peng AW, Ricci AJ, Heller S (2010). Mechanosensitive hair cell-like cells from embryonic and induced pluripotent stem cells. Cell.

[bib105] Ostrowski SM, Wright MC, Bolock AM, Geng X, Maricich SM (2015). Ectopic Atoh1 expression drives merkel cell production in embryonic, postnatal and adult mouse epidermis. Development.

[bib106] Ozaki H, Nakamura K, Funahashi J, Ikeda K, Yamada G, Tokano H, Okamura H, Kitamura K, Muto S, Kotaki H (2004). Six1 controls patterning of the mouse otic vesicle. Development.

[bib107] Pak AK, Slepecky NB (1995). Cytoskeletal and calcium-binding proteins in the mammalian organ of Corti: cell type-specific proteins displaying longitudinal and radial gradients. Hearing Research.

[bib108] Pan B, Géléoc GS, Asai Y, Horwitz GC, Kurima K, Ishikawa K, Kawashima Y, Griffith AJ, Holt JR (2013). TMC1 and TMC2 are components of the mechanotransduction channel in hair cells of the mammalian inner ear. Neuron.

[bib109] Phan D, Wodarz D (2015). Modeling multiple infection of cells by viruses: challenges and insights. Mathematical Biosciences.

[bib110] Qian D, Radde-Gallwitz K, Kelly M, Tyrberg B, Kim J, Gao WQ, Chen P (2006). Basic helix-loop-helix gene Hes6 delineates the sensory hair cell lineage in the inner ear. Developmental Dynamics.

[bib111] Rhee C, Lee BK, Beck S, LeBlanc L, Tucker HO, Kim J (2017). Mechanisms of transcription factor-mediated direct reprogramming of mouse embryonic stem cells to trophoblast stem-like cells. Nucleic Acids Research.

[bib112] Richardson GP, Forge A, Kros CJ, Fleming J, Brown SD, Steel KP (1997). Myosin VIIA is required for aminoglycoside accumulation in cochlear hair cells. The Journal of Neuroscience.

[bib113] Richardson GP, Forge A, Kros CJ, Marcotti W, Becker D, Williams DS, Thorpe J, Fleming J, Brown SD, Steel KP (1999). A missense mutation in myosin VIIA prevents aminoglycoside accumulation in early postnatal cochlear hair cells. Annals of the New York Academy of Sciences.

[bib114] Richardson RT, Atkinson PJ (2015). Atoh1 gene therapy in the cochlea for hair cell regeneration. Expert Opinion on Biological Therapy.

[bib115] Roberson DW, Rubel EW (1994). Cell division in the gerbil cochlea after acoustic trauma. The American Journal of Otology.

[bib116] Robinson JT, Thorvaldsdóttir H, Winckler W, Guttman M, Lander ES, Getz G, Mesirov JP (2011). Integrative genomics viewer. Nature Biotechnology.

[bib117] Roccio M, Hahnewald S, Perny M, Senn P (2015). Cell cycle reactivation of cochlear progenitor cells in neonatal FUCCI mice by a GSK3 small molecule inhibitor. Scientific Reports.

[bib118] Ronaghi M, Nasr M, Ealy M, Durruthy-Durruthy R, Waldhaus J, Diaz GH, Joubert LM, Oshima K, Heller S (2014). Inner ear hair cell-like cells from human embryonic stem cells. Stem Cells and Development.

[bib119] Ross SE, Greenberg ME, Stiles CD (2003). Basic helix-loop-helix factors in cortical development. Neuron.

[bib120] Ruben RJ, Sidman RL (1967). Serial section radioautography of the inner ear: histological technique. Archives of Otolaryngology - Head and Neck Surgery.

[bib121] Ryals BM, Rubel EW (1988). Hair cell regeneration after acoustic trauma in adult Coturnix quail. Science.

[bib122] Ryan AF, Ikeda R, Masuda M (2015). The regulation of gene expression in hair cells. Hearing Research.

[bib123] Sagwa EL, Ruswa N, Mavhunga F, Rennie T, Leufkens HG, Mantel-Teeuwisse AK (2015). Comparing amikacin and kanamycin-induced hearing loss in multidrug-resistant tuberculosis treatment under programmatic conditions in a namibian retrospective cohort. BMC Pharmacology and Toxicology.

[bib124] Sahly I, El-Amraoui A, Abitbol M, Petit C, Dufier JL (1997). Expression of myosin VIIA during mouse embryogenesis. Anatomy and Embryology.

[bib125] Sanyal A, Lajoie BR, Jain G, Dekker J (2012). The long-range interaction landscape of gene promoters. Nature.

[bib126] Scheffer DI, Shen J, Corey DP, Chen ZY (2015). Gene expression by mouse inner ear hair cells during development. Journal of Neuroscience.

[bib127] Shi Y, Lin S, Staats KA, Li Y, Chang WH, Hung ST, Hendricks E, Linares GR, Wang Y, Son EY, Wen X, Kisler K, Wilkinson B, Menendez L, Sugawara T, Woolwine P, Huang M, Cowan MJ, Ge B, Koutsodendris N, Sandor KP, Komberg J, Vangoor VR, Senthilkumar K, Hennes V, Seah C, Nelson AR, Cheng TY, Lee SJ, August PR, Chen JA, Wisniewski N, Hanson-Smith V, Belgard TG, Zhang A, Coba M, Grunseich C, Ward ME, van den Berg LH, Pasterkamp RJ, Trotti D, Zlokovic BV, Ichida JK (2018). Haploinsufficiency leads to neurodegeneration in C9ORF72 ALS/FTD human induced motor neurons. Nature Medicine.

[bib128] Sijacic P, Bajic M, McKinney EC, Meagher RB, Deal RB (2018). Changes in chromatin accessibility between Arabidopsis stem cells and mesophyll cells illuminate cell type-specific transcription factor networks. The Plant Journal.

[bib129] Singhal PK, Sassi S, Lan L, Au P, Halvorsen SC, Fukumura D, Jain RK, Seed B (2016). Mouse embryonic fibroblasts exhibit extensive developmental and phenotypic diversity. PNAS.

[bib130] Son EY, Ichida JK, Wainger BJ, Toma JS, Rafuse VF, Woolf CJ, Eggan K (2011). Conversion of mouse and human fibroblasts into functional spinal motor neurons. Cell Stem Cell.

[bib131] Stojanova ZP, Kwan T, Segil N (2016). Epigenetic regulation of *Atoh1* guides hair cell development in the mammalian cochlea. Development.

[bib132] Stone JS, Cotanche DA (2007). Hair cell regeneration in the avian auditory epithelium. The International Journal of Developmental Biology.

[bib133] Subramanian A, Tamayo P, Mootha VK, Mukherjee S, Ebert BL, Gillette MA, Paulovich A, Pomeroy SL, Golub TR, Lander ES, Mesirov JP (2005). Gene set enrichment analysis: a knowledge-based approach for interpreting genome-wide expression profiles. PNAS.

[bib134] Suzuki J, Hashimoto K, Xiao R, Vandenberghe LH, Liberman MC (2017). Cochlear gene therapy with ancestral AAV in adult mice: complete transduction of inner hair cells without cochlear dysfunction. Scientific Reports.

[bib135] Takahashi K, Tanabe K, Ohnuki M, Narita M, Ichisaka T, Tomoda K, Yamanaka S (2007). Induction of pluripotent stem cells from adult human fibroblasts by defined factors. Cell.

[bib136] Takahashi K, Yamanaka S (2006). Induction of pluripotent stem cells from mouse embryonic and adult fibroblast cultures by defined factors. Cell.

[bib137] Takebayashi S, Yamamoto N, Yabe D, Fukuda H, Kojima K, Ito J, Honjo T (2007). Multiple roles of notch signaling in cochlear development. Developmental Biology.

[bib138] Tan F, Chu C, Qi J, Li W, You D, Li K, Chen X, Zhao W, Cheng C, Liu X, Qiao Y, Su B, He S, Zhong C, Li H, Chai R, Zhong G (2019). AAV-ie enables safe and efficient gene transfer to inner ear cells. Nature Communications.

[bib139] Tao Y, Huang M, Shu Y, Ruprecht A, Wang H, Tang Y, Vandenberghe LH, Wang Q, Gao G, Kong WJ, Chen ZY (2018). Delivery of Adeno-Associated virus vectors in adult mammalian Inner-Ear cell subtypes without auditory dysfunction. Human Gene Therapy.

[bib140] Tarchini B, Tadenev AL, Devanney N, Cayouette M (2016). A link between planar polarity and staircase-like bundle architecture in hair cells. Development.

[bib141] Treutlein B, Lee QY, Camp JG, Mall M, Koh W, Shariati SA, Sim S, Neff NF, Skotheim JM, Wernig M, Quake SR (2016). Dissecting direct reprogramming from fibroblast to neuron using single-cell RNA-seq. Nature.

[bib142] Troutt LL, van Heumen WR, Pickles JO (1994). The changing microtubule arrangements in developing hair cells of the chick cochlea. Hearing Research.

[bib143] Vaisbuch Y, Santa Maria PL (2018). Age-Related hearing loss: innovations in hearing augmentation. Otolaryngologic Clinics of North America.

[bib144] Van Pham P, Vu NB, Dao TT, Le HT, Phi LT, Phan NK (2017). Production of endothelial progenitor cells from skin fibroblasts by direct reprogramming for clinical usages. In Vitro Cellular & Developmental Biology - Animal.

[bib145] van Vliet E, Daneshian M, Beilmann M, Davies A, Fava E, Fleck R, Julé Y, Kansy M, Kustermann S, Macko P (2014). Current approaches and future role of high content imaging in safety sciences and drug discovery. ALTEX.

[bib146] Villanueva-Paz M, Povea-Cabello S, Villalón-García I, Suárez-Rivero JM, Álvarez-Córdoba M, de la Mata M, Talaverón-Rey M, Jackson S, Sánchez-Alcázar JA (2019). Pathophysiological characterization of MERRF patient-specific induced neurons generated by direct reprogramming. Biochimica Et Biophysica Acta (BBA) - Molecular Cell Research.

[bib147] Volpi EV, Chevret E, Jones T, Vatcheva R, Williamson J, Beck S, Campbell RD, Goldsworthy M, Powis SH, Ragoussis J, Trowsdale J, Sheer D (2000). Large-scale chromatin organization of the major histocompatibility complex and other regions of human chromosome 6 and its response to interferon in interphase nuclei. Journal of Cell Science.

[bib148] Wallis D, Hamblen M, Zhou Y, Venken KJ, Schumacher A, Grimes HL, Zoghbi HY, Orkin SH, Bellen HJ (2003). The zinc finger transcription factor Gfi1, implicated in Lymphomagenesis, is required for inner ear hair cell differentiation and survival. Development.

[bib149] Wang J, Mark S, Zhang X, Qian D, Yoo SJ, Radde-Gallwitz K, Zhang Y, Lin X, Collazo A, Wynshaw-Boris A, Chen P (2005). Regulation of polarized extension and planar cell polarity in the cochlea by the vertebrate PCP pathway. Nature Genetics.

[bib150] Wapinski OL, Vierbuchen T, Qu K, Lee QY, Chanda S, Fuentes DR, Giresi PG, Ng YH, Marro S, Neff NF, Drechsel D, Martynoga B, Castro DS, Webb AE, Südhof TC, Brunet A, Guillemot F, Chang HY, Wernig M (2013). Hierarchical mechanisms for direct reprogramming of fibroblasts to neurons. Cell.

[bib151] Wapinski OL, Lee QY, Chen AC, Li R, Corces MR, Ang CE, Treutlein B, Xiang C, Baubet V, Suchy FP, Sankar V, Sim S, Quake SR, Dahmane N, Wernig M, Chang HY (2017). Rapid chromatin switch in the direct reprogramming of fibroblasts to neurons. Cell Reports.

[bib152] White PM, Doetzlhofer A, Lee YS, Groves AK, Segil N (2006). Mammalian cochlear supporting cells can divide and trans-differentiate into hair cells. Nature.

[bib153] WHO (2019). World health organization. https://www.who.int.

[bib154] Wong AC, Ryan AF (2015). Mechanisms of sensorineural cell damage, death and survival in the cochlea. Frontiers in Aging Neuroscience.

[bib155] Woods C, Montcouquiol M, Kelley MW (2004). Math1 regulates development of the sensory epithelium in the mammalian cochlea. Nature Neuroscience.

[bib156] Wu Z, Grillet N, Zhao B, Cunningham C, Harkins-Perry S, Coste B, Ranade S, Zebarjadi N, Beurg M, Fettiplace R, Patapoutian A, Mueller U (2017). Mechanosensory hair cells express two molecularly distinct mechanotransduction channels. Nature Neuroscience.

[bib157] Xu J, Du Y, Deng H (2015). Direct lineage reprogramming: strategies, mechanisms, and applications. Cell Stem Cell.

[bib158] Yang J, Cong N, Han Z, Huang Y, Chi F (2013). Ectopic hair cell-like cell induction by Math1 mainly involves direct transdifferentiation in neonatal mammalian cochlea. Neuroscience Letters.

[bib159] Yoon SJ, Elahi LS, Pașca AM, Marton RM, Gordon A, Revah O, Miura Y, Walczak EM, Holdgate GM, Fan HC, Huguenard JR, Geschwind DH, Pașca SP (2019). Reliability of human cortical organoid generation. Nature Methods.

[bib160] Yoshimura H, Shibata SB, Ranum PT, Moteki H, Smith RJH (2019). Targeted allele suppression prevents progressive hearing loss in the mature murine model of human TMC1 deafness. Molecular Therapy.

[bib161] Zhang W, Morris QD, Chang R, Shai O, Bakowski MA, Mitsakakis N, Mohammad N, Robinson MD, Zirngibl R, Somogyi E, Laurin N, Eftekharpour E, Sat E, Grigull J, Pan Q, Peng WT, Krogan N, Greenblatt J, Fehlings M, van der Kooy D, Aubin J, Bruneau BG, Rossant J, Blencowe BJ, Frey BJ, Hughes TR (2004). The functional landscape of mouse gene expression. Journal of Biology.

[bib162] Zhang T, Xu J, Maire P, Xu PX (2017). Six1 is essential for differentiation and patterning of the mammalian auditory sensory epithelium. PLOS Genetics.

[bib163] Zheng W, Huang L, Wei ZB, Silvius D, Tang B, Xu PX (2003). The role of Six1 in mammalian auditory system development. Development.

[bib164] Zheng JL, Gao WQ (2000). Overexpression of Math1 induces robust production of extra hair cells in postnatal rat inner ears. Nature Neuroscience.

[bib165] Zhu Y, Scheibinger M, Ellwanger DC, Krey JF, Choi D, Kelly RT, Heller S, Barr-Gillespie PG (2019). Single-cell proteomics reveals changes in expression during hair-cell development. eLife.

